# Tailoring the Solvation of Aqueous Zinc Electrolytes by Balancing Kosmotropic and Chaotropic Ions

**DOI:** 10.1021/acsnano.4c16521

**Published:** 2025-02-06

**Authors:** Ibrahim Al Kathemi, Zaher Slim, Fernando Igoa Saldaña, Ann-Christin Dippel, Patrik Johansson, Mateusz Odziomek, Roza Bouchal

**Affiliations:** † Department of Colloid Chemistry, Max Planck Institute of Colloids and Interfaces, Am Mühlenberg 1, 14476 Potsdam, Germany; ‡ Department of Physics, Chalmers University of Technology, 41296 Gothenburg, Sweden; § Deutsches Elektronen-Synchrotron DESY, Notkestraße 85, 22607 Hamburg, Germany; ∥ Alistore-European Research Institute, CNRS FR 3104, Hub de I’Energie, Rue Baudelocque, 80039 Amiens, France

**Keywords:** weakly solvated electrolyte, aqueous eutectic electrolyte, chaotropic ions, kosmotropic ions, zinc solvation structure, water coordination

## Abstract

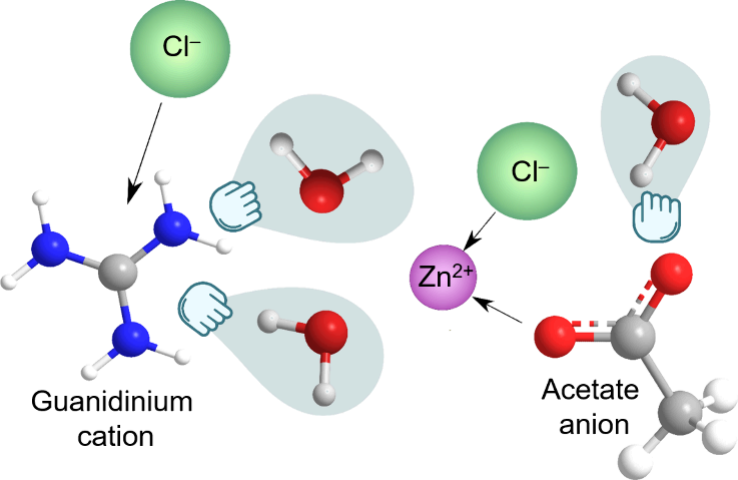

Aqueous zinc (Zn) batteries (AZBs) have emerged as a highly promising concept for grid-scale electrochemical energy storage due to the prospects of high safety, low cost, and competitive energy density. However, the commonly employed electrolytes, at ca. 0.5–2 M salt concentration, significantly limit the cycling stability due to the uncontrolled hydrogen evolution reaction (HER). This originates from the plentiful access of free water molecules that become hydrolyzed. As a remedy, highly concentrated electrolytes, ca. 10 m and higher, have been suggested by means of altering the local solvation, promoting Zn^2+^–anion rather than Zn^2+^–H_2_O coordination, but this renders high viscosity electrolytes with reduced ion transport. Here, by balancing a combination of kosmotropic and chaotropic ions, specifically acetate (Ac) and guanidinium (Gua), it is possible to tailor their strong and weak coordination with water, respectively. This strategy results in a weakly solvated electrolyte with improved ion transport properties alongside stabilization of the Zn metal anode. Furthermore, our electrolyte also enhances the cathode stability, rendering an overall increase in the battery lifetime and performance. Hence, this electrolyte design strategy can be applied to the development of a new generation of AZBs.

## 1 Introduction

The development of sustainable energy storage technologies is needed for grid integration of renewable and clean energies, such as wind and solar power.^1−4^ Finding a battery technology that can fulfill the quite demanding requirements of safety, cost efficiency, and long-term stability is therefore of utmost importance.^5,6^ Aqueous zinc (Zn) batteries (AZBs) are considered promising for such large-scale applications, as Zn is an abundant noncritical raw material,^7^ relatively inexpensive (as an example, in recent years battery grade Li_2_CO_3_ was rated at between 5.8 and 80 USD/kg, whereas Zn as commodity metal was at a mere 1.85 to 4.4 USD/kg),^8^ and also easy to recover and recycle.^9^ Furthermore, AZBs use safe, nontoxic, nonflammable aqueous electrolytes,^10−14^ and the metal anode possesses an attractive specific capacity (≈820 mAh/g).^15,16^ However, rechargeable AZBs are still far from fulfilling the requirements, with many challenges remaining related to cycling stability, especially at the zinc anode side, due to dendrite formation,^17^ parasitic hydrogen evolution reaction (HER),^18^ corrosion,^19^ and passivation.^20^

Designing new highly concentrated electrolytes (HCEs) has recently been considered the most promising and cost-effective strategy to overcome these challenges.^21−23^ Overall, the main goal has been to deplete the Zn^2+^ first solvation shell from water, e.g., by increasing the Zn-salt concentration, and thereby achieve reduced water activity.^24,25^ Such HCEs have become the most facile strategy for suppressing the HER, but this comes at the expense of high viscosity,^26^ which leads to impeded ion transport.^27^ Alternatively, water can be partially replaced in the first solvation shell by introducing acetonitrile,^28,29^ propylene carbonate,^30,31^ dimethylformamide,^32,33^ and ether-based (dioxolane^34,35^ and glymes)^36^ solvents to create hybrid electrolytes, which considerably improves the Zn metal anode stability. However, some of the aforementioned chemicals are classified as carcinogenic, mutagenic, and reprotoxic (CMR). The use of CMR chemicals in high concentrations (up to 60%) contradicts the principles of safety and sustainability in the design of aqueous electrolytes and consequently in AZBs as well.

Our design strategy is therefore instead based on a careful selection of salts/ions with specific influence on the electrolyte structure, using the “kosmotropes”, structure making, and “chaotropes”, structure breaking, classification and the well-established Hofmeister series as guidance.^37,38^ Kosmotropes create strongly hydrated solutes and hence increase the order, while chaotropes are weakly hydrated and render smaller changes in the viscosity by decreasing the order of water.^39^ In the context of Zn-based electrolytes, Zn^2+^ is characterized as a kosmotropic cation with strong coordination with water, which is the underlying cause of the observed ample hydrolysis. Kosmotropic anions, such as formate, acetate (Ac), and NO_3_^–^ can all compete with Zn^2+^ and interact with water.^23,40,41^ This approach was recently employed using Ac for Zn^2+^, K^+^, and Na^+^-based electrolytes to alter the water coordination.^42−44^ On the other hand, chaotropic anions,^45−48^ in particularly perchlorate, are commonly used to reduce the amount of free water in highly concentrated electrolytes. However, at high concentrations, perchlorates can pose a risk of toxicity and battery explosion.^49^ In contrast, chaotropic cations like ammonium,^50^ tetramethylammonium,^51^ and guanidinium (Gua) have received less attention in Zn aqueous electrolytes. Hence, the impact of the collective properties of both kosmotropic and chaotropic ions within the same electrolyte system remains largely unexplored.

In this work, we combine for the first time a strongly chaotropic cation, Gua, with a strongly kosmotropic anion, Ac, to find the right balance to prepare a weakly solvating electrolyte. Our hypothesis is that Ac coordinates both Zn^2+^ and water, while Gua coordinates with water and induces disorder. We probe this by mixing ZnCl_2_ and GuaAc salts in different ratios. By employing physicochemical, structural, and electrochemical characterization techniques, alongside with density functional theory (DFT) calculations, the solvation structure of the Zn^2+^ ions was assessed. To further evaluate the practical applicability of the changed solvation structure, a selected electrolyte was tested in a full AZB laboratory cell, allowing for a comprehensive assessment of its electrochemical performance.

## 2 Results and Discussion

### 2.1 Electrolyte Composition and Solvation Structure

**Figure 1:**
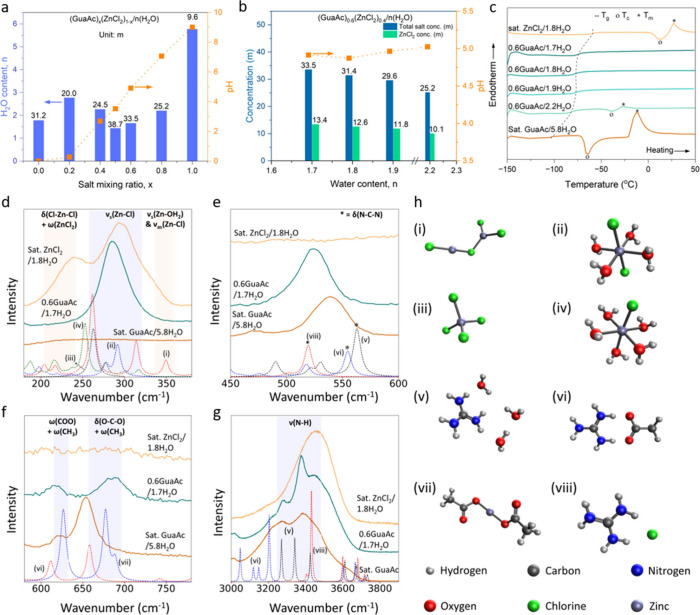
Compositional and local structure characterization of the GuaAc/ZnCl_2_ electrolytes. (a) Total salt concentration, water content, and pH of the chosen salt mixing ratios. (b) Total salt and ZnCl_2_ concentration, water content, and pH of the 0.6GuaAc-based electrolytes. (c) DSC analysis of the chosen electrolytes. (d–h) Experimental and calculated (dashed trace) Raman spectra corresponding to each species.

The electrolyte formulation was optimized by mixing ZnCl_2_ and GuaAc at different ratios to saturation in water. Saturation concentrations were used to minimize the number of free water and to strengthen the bond between anions and cations with water, thus suppressing unwanted side reactions and increasing the electrochemical stability window.^52^ The mixtures are denoted (GuaAc)_*x*_(ZnCl_2_)_1–*x*_/*n*(H_2_O) in the figures, where *x* (and 1 – *x*) is the internal salt molar ratio and *n* is the mol of water per total mol of salt. For simplicity, “*x*GuaAc” will be used throughout the text. Introducing GuaAc renders higher salt saturation concentrations than saturated ZnCl_2_ (sat. ZnCl_2_), at ca. 0.5GuaAc (Figure 1a, Table S1), which is due to decreased and more distorted hydrogen bonding (HB).^53^ This is mostly because the maximum concentration of ZnCl_2_ and GuaAc is reached around those ratios (Table S1). This suggests that at these ratios, the stability between the two salts optimally balances the hydrogen bonding within the mixture. This approach takes advantage of the high ZnCl_2_ solubility and the chaotropic nature of the Gua cation to disrupt hydrogen bonds, as well as its potential hydrotropic effect within the mixture. This hydrotropic effect enhances solubility through the formation of soluble complexes/double salts/associations with hydrotropic solubilization agents.^54^ By increasing the GuaAc concentration, the mixed electrolyte pH moves from very acidic (pH = 0.3) through mildly acidic and neutral pH to finally an alkaline pH in the sat. GuaAc electrolytes (pH = 9). Furthermore, aging for 60 days at room temperature revealed the formation of crystals and/or phase separation in all electrolytes except the sat. GuaAc and 0.6GuaAc (Figure S1a). Taking into consideration the above, including the Zn^2+^ concentration, the 0.6GuaAc electrolyte was selected for further optimization (Figure 1b, Table S2). A minor increase in pH was obtained as a function of increased water concentration, but the mildly acidic environment was maintained, and aging studies also confirmed these electrolytes to be stable (Figure S1b).

To understand the impact of GuaAc and water concentration in GuaAc/ZnCl_2_ electrolytes, 0.6GuaAc, its diluted forms (increase in *n*), 0.2GuaAc and 0.8GuaAc, as well as sat. ZnCl_2_ and sat. GuaAc were analyzed. Differential scanning calorimetry (DSC) revealed that all mixing ratios had glass transition temperatures (*T*_g_) lower than that of sat. ZnCl_2_ (Table S3). The 0.2GuaAc and 0.6GuaAc electrolytes showed characteristics of eutectic mixtures, with suppressed melting (*T*_m_) and crystallization (*T*_c_) temperatures (Figure S2).^55^ However, increasing the water concentration in 0.6GuaAc from *n* = 1.9 to *n* = 2.2 led to significant structural changes, as *T*_m_ and *T*_c_ were detected in the most diluted 0.6GuaAc electrolyte (Figure 1c, Table S4).

To gain more insight into the local electrolyte structure, experimental Raman spectra were compared with the DFT-computed spectra of potential species (Table S5). The DFT calculations suggest that the peak present in the Zn^2+^–Cl^–^ and Zn^2+^–H_2_O region of the 0.6GuaAc electrolyte spectra (200–450 cm^–1^) can be assigned to Zn–Cl symmetric bond stretch vibrations, comprising various Zn–Cl species, including but not limited to structures (i), (ii), (iii), and (iv) (Figure 1d–h, Figure S3a). Additionally, this region appears identical to that of a less concentrated (<20 m) ZnCl_2_ electrolyte,^26,56^ suggesting that higher ZnCl_2_ aggregates such as (i) do not exist in the optimized 0.6GuaAc. Additionally, this peak shifts to higher wavenumbers with increasing ZnCl_2_ concentration, indicating that due to GuaAc, the solvation shell of Zn^2+^ is (partially) dehydrated. Also, the peaks of [ZnCl_4_]^2–^ and ZnCl^+^ oligomers at 243 and 342 cm^–1^ respectively,^42,57^ are not present in the mixed GuaAc/ZnCl_2_ electrolytes, even at lower concentrations of GuaAc. On the other hand, there is no significant change in the 0.6GuaAc Raman spectra upon increasing the water content (Figure S4a). Based on these results, the proposed species profile for ZnCl_2_ in water electrolyte is governed by equilibria 1 and 2:
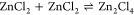
1
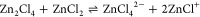
2

The water-retaining effect of Gua (v) and its interaction with Ac ions (vi) and Cl^–^ (viii) can be inferred by tracking the shifts in the N–C–N bending mode in the range 400–1500 cm^–1^^58−60^ (Figure S3b,c), from 541 cm^–1^ (sat. GuaAc) to 523 cm^–1^ (0.6GuaAc), attributed to the formation of GuaCl through the following reaction:
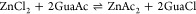
3

To further support this, the region at 600–800 cm^–1^ and the spectra of the sat. GuaAc were compared to those of mixed electrolytes, wherein the peaks emerging at 616 and 670 cm^–1^ are attributed to the formation of a ZnAc_2_ complex (vii).^59^ Furthermore, shifts to higher frequencies can be found for the C–C stretching, CH_3_ bending, and C=O stretching modes, confirming the preference of Ac to coordinate with Zn^2+^. According to the literature and the DFT calculations, these peaks in sat. GuaAc are assigned to free Ac, while the shift to higher frequencies in GuaAc/ZnCl_2_ electrolytes indicates the formation of Zn^2+^–Ac interactions.^58^ In addition, the two peaks at 1562 and 1662 cm^–1^ can both be assigned to a Gua H–N–H bending mode,^61,62^ which also both decrease in intensity with lower GuaAc content. There are no significant shifts in the Ac Raman spectra for the 0.6GuaAc electrolytes with increasing water content (Figure S4b,c). Additionally, the DFT-calculated binding energy for various Zn complexes (Table S6) renders the Zn[Ac]_2_·4H_2_O species the most stable, suggesting that Zn^2+^ has a preference for Ac. Overall, the Raman analysis strongly suggests that a double displacement reaction occurs at specific ZnCl_2_ to GuaAc in water ratios. To confirm reaction 3, a solution composed of 1 mol of ZnAc_2_ and 2 mol of GuaCl was prepared, corresponding to (ZnAc_2_)_0.33_(GuaCl)_0.67_/1.6H_2_O (Table S7). The obtained solution remained homogeneous and stable at both −18 and −80 °C, as can be seen in Figure S5, indicating that a eutectic electrolyte was formed.

To investigate the impact of Gua and Ac on the water environment, the 2800–3800 cm^–1^ range (Figures S3d and S4d), corresponding to the N–H and the O–H stretching vibrations, was deconvoluted (Figure S6), and the contributions were categorized into strong (peak 1), weak (peak 2), and non-HB (peak 3).^63,64^ In sat. GuaAc, two additional peaks for the N–H stretching vibration were observed, with peak 4 attributed to Gua–Ac interactions and peak 5 to Gua–water interactions. The OH vibration peaks shift more than the N–H vibration peak, and the HB peak frequencies in 0.2GuaAc are similar to those in sat. ZnCl_2_. For the peak areas in 0.2GuaAc (Figure S7), the weak and non-HB peaks increased, while the strong HB peak decreased, likely indicating ZnCl_2_–H_2_O interactions for lower GuaAc content. The strong HB peak area percentage is lower in mixed electrolytes than in sat. ZnCl_2_. Overall, Raman results show that GuaAc suppresses the O–H vibrations, confirming the coordination of Gua with water rather than with Zn^2+^. For the N–H stretching, the Gua–Ac peak in sat. GuaAc shifts to higher wavenumbers in 0.6GuaAc, while the peak area percentage drops from 30% in sat. GuaAc to 10% in GuaAc/ZnCl_2_ (Figure S7b), confirming the preference of Zn^2+^ for Ac. The fact that peak 5 has nearly twice the area in the GuaAc/ZnCl_2_ mixtures, with a maximum for 0.6GuaAc, is attributed to increased Gua–water interactions in the eutectic electrolyte. Combined with that, the Gua–Cl peak shifts to higher frequencies moving from 0.8GuaAc to 0.2GuaAc, with a slight decrease in area as the ZnCl_2_ concentration increases, suggest that Cl^–^ prefers coordinating Gua over Zn^2+^ in 0.2GuaAc, in agreement with our DFT calculations (eq 3). Finally, in the diluted 0.6GuaAc electrolytes, there are significant peak shifts only in the water region (2800–3800 cm^–1^) of the 0.6GuaAc/1.9H_2_O electrolyte (Figure S6c): the Gua–Ac peak shifts to higher wavenumbers, the non-HB peak to lower wavenumbers, and both peak areas increase, which indicates that even a slight variation in water concentration impacts the Ac and water coordination, confirming the formation of a stable eutectic aqueous ternary mixture.

Synchrotron X-ray total scattering provides insights into the ion–ion interactions, and via the Fourier analysis of the scattered intensity, the pair distribution function (PDF) (Figure S8) shows a peak at 2.2 Å to be present in all electrolytes containing ZnCl_2_, which is therefore assigned to Zn–Cl,^65^ consistent with our DFT-calculated distance of 2.31 Å (Figure S9). The peak at 2.8 Å is attributed to O–O in water, which is present in all electrolytes, but in sat. GuaAc, the intensity is significantly higher due to the presence of more water. The DFT-calculated Zn–Zn distance for structure (i) and the Cl–Cl distance for structure (iii) are 3.74 and 3.78 Å (Figure S9), respectively, and correspond to the peak observed at ca. 3.9 Å in Figure S8. This peak is found only for the sat. ZnCl_2_ electrolyte and is attributed to ZnCl_2_-dimers and/or higher aggregates.^57^ This suggests that higher ZnCl_2_ aggregates such as (i) do not exist in the optimized 0.6GuaAc electrolyte, which is consistent with the DFT calculations. Additionally, partial PDF patterns for Gua were computed (Figure S10), and these further support the peak assignments made. This observation could explain the high viscosity and low ionic conductivity of sat. ZnCl_2_ (below) and indicates that the addition of GuaAc weakened the Zn–Zn and Cl–Cl interactions, which is thus in agreement with the Raman spectra analysis. The impact of water concentration in the 0.6Gua electrolytes is reflected in the PDF patterns by a peak shift representing the C–N bond length but with low correlation to the water content.

By combining a kosmotropic anion (Ac) and a chaotropic cation (Gua) in the preparation of a Zn aqueous electrolyte, we demonstrated the possibility of efficiently altering the Zn solvation shell. At an optimized ratio of 0.6GuaAc:0.4ZnCl_2_ and *n* = 1.9 water ratio, the obtained electrolyte demonstrated an increase in pH, the formation of an eutectic electrolyte, and a shift of Zn^2+^–water coordination to Zn^2+^–Ac and Gua–water coordination.

### 2.2 Electrolyte Transport Properties

**Figure 2:**
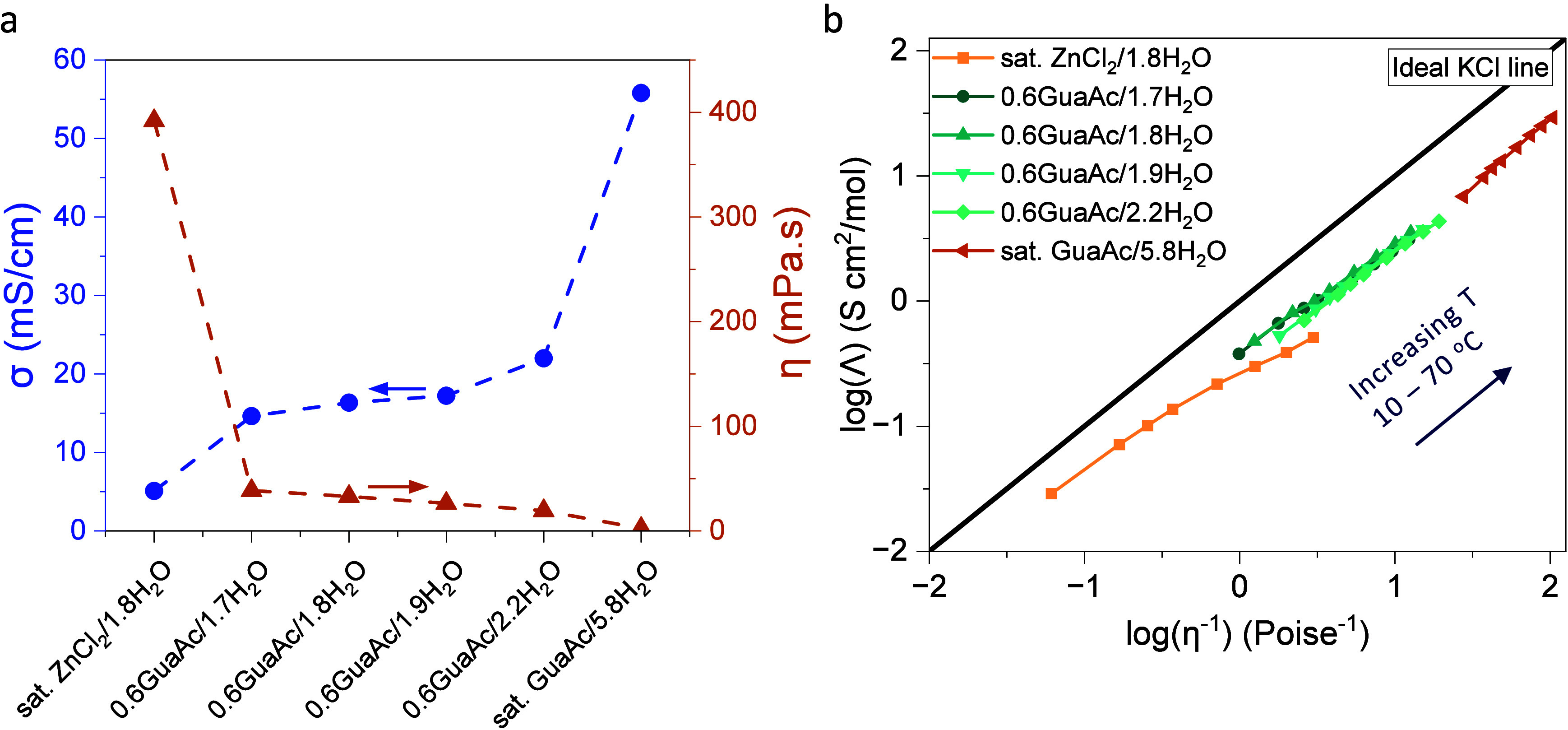
Transport properties of sat. ZnCl_2_, sat. GuaAc, and 0.6GuaAc-based electrolytes. (a) Ion conductivity and viscosity at 25 °C. (b) Walden plot in the temperature range 10 to 70 °C.

The ionic conductivity (σ), viscosity (η), and density (ρ) (Figure S11) of the optimized 0.6GuaAc electrolytes at various water contents indicate enhanced transport properties as compared to those of sat. ZnCl_2_. At 25 °C (Figure 2a) the ionic conductivity increases from 5 mS/cm for sat. ZnCl_2_ to 22 mS/cm for 0.6GuaAc/2.2H_2_O, while the viscosity decreases significantly, from 392 mPa·s in sat. ZnCl_2_ to 39 mPa·s in 0.6GuaAc/1.7H_2_O and even more with higher water content, which is attributed to a more disordered water structure.^66^

The temperature dependence of the ionic conductivity and the viscosity could in general follow either Arrhenius (Figure S12) or Vogel–Fulcher–Tammann (VFT, eq S1, Figure S13) behavior,^67,68^ and here the absence of a linear correlation with 1/*T* in the Arrhenius model indicates the latter. Furthermore, the difference between the theoretical glass transition *T*_0_^*i*^ and the experimental *T*_g_ is dependent on the fragility and strength of the liquid,^69^ and here *T*_0_^σ^ differs only by a maximum of 5 K, while _*T*_0_^η^_ differs by −15 K for 0.6GuaAc/1.7H_2_O and 0.6GuaAc/1.8H_2_O (Tables S8 and S9). This indicates that the electrolytes are more fragile, displaying significant changes in transport properties close to *T*_g_. Likewise, the activation energy (*E*_a_^*i*^, Tables S8 and S9) shows *E*_a_^σ^ values very similar for all electrolytes, but sat. ZnCl_2_ exhibits a larger *E*_a_^*i*^. The difference between the ideal *T*_g_ and the measured *T*_g_ and the *E*_a_^*i*^ reported in this paper are in the same order of magnitude as those in the literature for LiCl/ZnCl^70^ and Zn(TFSI)_2_^71^ electrolytes.

Furthermore, the ionicity was assessed by using Walden plots (eq S2 and Figure 2b) with a correction factor, following Yang et al.^70^ All electrolytes display approximately the same ionicity, >90%. However, with increasing *T*, the ionicity of sat. ZnCl_2_ decreases, while it does not change for the 0.6GuaAc-based electrolytes. The decrease in ionicity can be due to more correlated ion motion by ion pairing.^70^

Finally, the transport number of Zn^2+^ ions (*t*_Zn^2+^_, Figure S14), determined using the Bruce–Vincent method,^72,73^ increases from 0.6 ± 0.01 for sat. ZnCl_2_ to 0.71 ± 0.01 for 0.6GuaAc/1.7H_2_O. However, the increased water content in 0.6GuaAc reduces the experimental stability for *n* = 1.8 and 2.2, causing larger error bars, and while not fully understood, it nevertheless confirms the importance of fine-tuning the water content.

### 2.3 Electrolyte Stability and Zn Plating and Stripping

Linear sweep voltammetry (LSV) voltammograms (Figure S15a) reveal that by mixing GuaAc with ZnCl_2_, the electrochemical stability window (ESW) expands. The reduction potential shift from −0.7 V for sat. ZnCl_2_ to −1 V vs Ag/AgCl for the 0.6GuaAc electrolyte (Figure S15b) is related to shifted HER. At positive potentials, the various 0.6GuaAc electrolytes show no significant differences, whereas sat. GuaAc has a lower limit, probably due to Ac oxidation, which suggests that the latter is suppressed in the 0.6GuaAc electrolytes.

**Figure 3:**
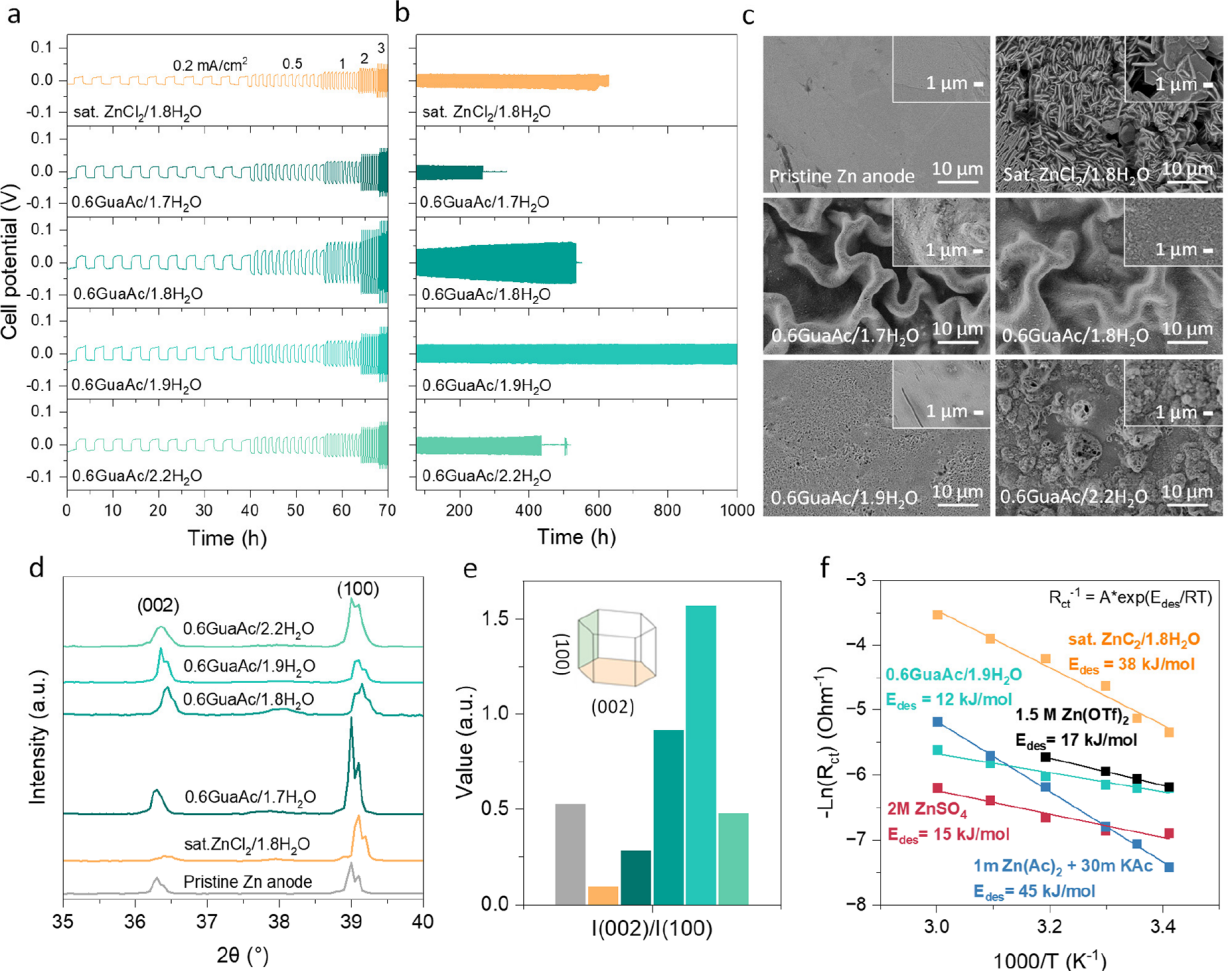
Electrochemical characterization of the GuaAc/ZnCl_2_ electrolytes. (a) Galvanostatic cycling at different rates and (b) long-term cycling at 0.5 mA/cm^2^ utilizing a Zn∥Zn cell with a capacity of 0.4 mAh/cm^2^. (c) SEM pictures of the pristine and recovered Zn foil treated with GuaAc-based electrolytes after applying 0.5 mA/cm^2^ with a capacity of 0.4 mAh/cm^2^ for 10 cycles ending with a discharge. (d) XRD patterns of the recovered Zn anodes of (c). (e) Ratios of *I*(002)/*I*(100). (f) Determined desolvation energy of dilute and highly concentrated electrolytes.

Moving to the Zn plating and stripping, this was initially evaluated using the modified Aurbach Coulombic efficiency method proposed by Vazquez et al.,^40^ whereby all electrolytes show a Coulombic efficiency of >90% (Figure S16), which is low compared to what has been reported for HCEs in the literature. However, using Zn_0.2_K_0.8_OAC_1.2_·10H_2_O of Vazquez et al.^40^ and Li_2_ZnCl_4_·9H_2_O of Yang et al.,^70^ we find them on par, highlighting the inconsistency in the AZB literature,^74^ due to methodological differences. Symmetric Zn∥Zn cells under galvanostatic conditions were subjected to both rate capability tests and long-term cycling (Figure 3a,b and Figure S17). The former revealed that all 0.6GuaAc electrolytes exhibit slightly higher overpotentials, 6–37 mV higher, than sat. ZnCl_2_, and the latter revealed that 0.6GuaAc/1.9H_2_O is the most stable electrolyte with >1000 h of cycling at 0.5 mA/cm^2^.

Furthermore, the selected 0.6GuaAc/1.9H_2_O was tested at a higher current density of 1 mA/cm^2^ (Figure S18a,b) while maintaining a constant capacity. The 0.6GuaAc/1.9H_2_O had stable cycling for over 1200 h, while sat. ZnCl_2_ cycled for only 500 h. Second, the capacity was increased to 2.5 mAh/cm^2^ (Figure S18c), with the current density held at 0.5 mA/cm^2^, to achieve a C/5 rate. Under these conditions, the 0.6GuaAc/1.9H_2_O had stability for over 500 h. These findings further support the feasibility and practical applicability of the 0.6GuaAc electrolyte in the relevant applications.

The surface morphology and composition of recovered Zn anodes were assessed by scanning electron microscopy (SEM), X-ray diffraction (XRD), and X-ray photoelectron spectroscopy (XPS). First, SEM images taken after 10 cycles at 0.5 mA/cm^2^ (Figure 3c, Figure S19) show Zn deposits forming hexagonal platelets that are predominantly arranged in a vertical pattern and distributed unevenly when using sat. ZnCl_2_. In stark contrast, the Zn anodes from the 0.6GuaAc/nH_2_O cells, for *n* = 1.7, show wrinkled Zn deposition that fades away with higher water content (*n* = 1.8) until a homogeneous Zn surface is observed for *n* = 1.9. Increasing the water content to *n* = 2.2, a rougher surface with bump-like shapes is demonstrated. These results are corroborated by the XRD patterns (Figure 3d) that display an increase in the (002) peak, the most stable facet with the lowest surface energy, with increasing water content up until *n* = 1.9 (Figure 3e).^75^ Although the 0.6GuaAc electrolytes are similar in composition, solvation structure, and transport properties, the 0.6GuaAc/1.9H_2_O is superior to the other water ratios. It is hypothesized that at lower water content (*n* = 1.7), Zn ions tend to form local clusters at the electrode interface, creating regions with a high local concentration of plated Zn. These regions then act as favorable sites for additional Zn clusters to plate, resulting in the wrinkle effect observed in Figure 3c. When the water content is slightly increased to *n* = 1.8, the availability of water molecules reduces this clustering effect. By *n* = 1.9, Zn ion clusters no longer form, leading to homogeneous Zn plating. However, at higher water content (*n* = 2.2), issues like hydrogen evolution and corrosion arise due to electrolyte structure changes, as indicated by DSC measurements (Figure 1c). Thus, adjusting the water content from *n* = 1.7 to 1.9 optimizes Zn plating uniformity. However, more research has to be conducted to prove the formation of Zn^2+^ clusters and to support this hypothesis.

Additionally, a peak at 37.9° is observed in some samples, attributed to residual ZnCl_2_ crystals from the electrolyte.^76^ Finally, the XPS analysis (Figure S20 and Table S10) shows the chemical composition at the surfaces to be similar. The spectra of the O 1s fit to two organic species and one ZnO/surface hydroxyl species, the latter being particularly strong for the pristine Zn anode, implying that the acidic electrolytes can etch the surface even without applying a current. For the N 1s spectra, amide (N—C(=O)), imine (C=N—C), and amine (R–NH_2_) constituents are associated with Gua. Deconvolution of the Cl 2p spectra is assigned to ionic Cl^–^, as in ZnCl_2_ and Gua/Cl^–^. Overall, the XPS data show the formation of an organic byproduct layer in the presence of GuaAc, which might contribute to the Zn surface stabilization.

Finally, to confirm the weakly solvating character of 0.6GuaAc/1.9H_2_O, the desolvation energy (*E*_des_, Figure 3f and Figure S21) was obtained from the Arrhenius equation.^77^ The 12 kJ/mol value of 0.6GuaAc/1.9H_2_O is significantly lower than the 38 kJ/mol value of sat. ZnCl_2_, suggesting enhanced zinc deposition kinetics. Furthermore, to compare the obtained data, we have measured the desolvation energy of three standard electrolytes: low concentration electrolytes, i.e., 2 M ZnSO_4_ and 1.5 M Zn(OTf)_2_, and a concentrated electrolyte consisting of 1 m Zn(Ac)_2_ + 30 m KAc.^54^ It can be concluded that 0.6GuaAc/1.9H_2_O has an *E*_des_ in the same range as those of diluted electrolytes but considerably lower than those of HCEs proposed in the literature. However, it is important to note that the values obtained for the standard diluted electrolytes deviate from those reported in the literature^36,78−86^ (Figure S21g). This highlights the significance of preparing and testing literature-based electrolytes in-house, ensuring that the experimental parameters are consistent and thereby allowing for a fair and accurate comparison.

### 2.4 Performance of Zn∥ZnVO Full Cells

**Figure 4:**
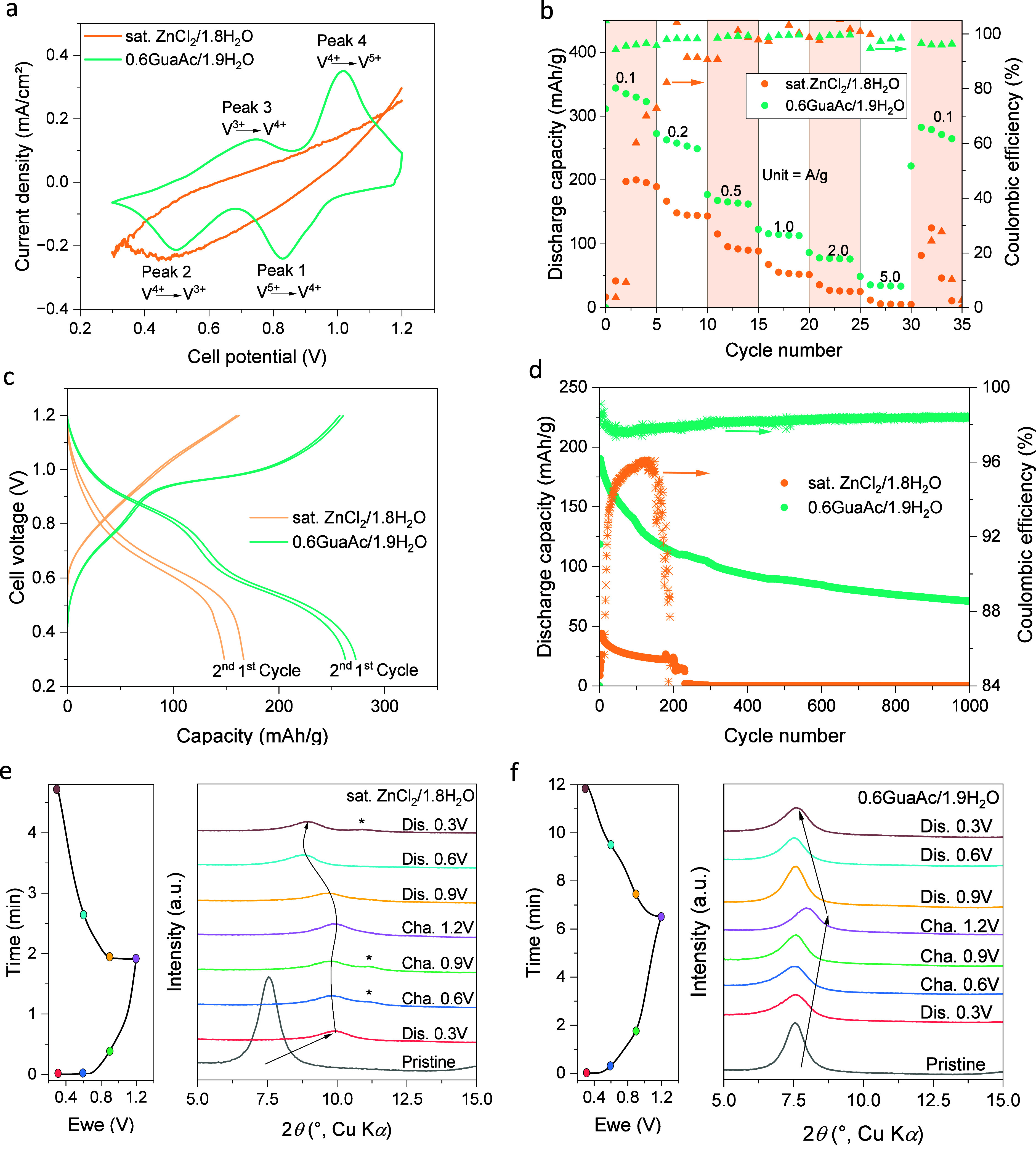
Full cell performance of the 0.6GuaAc/1.9H_2_O electrolyte compared to the sat. ZnCl_2_ electrolyte in a Zn∥ZnVO Swagelok cell. (a) CV profiles at a scan rate of 0.1 mV/s. (b) Rate capability at different current densities. (c) Cycling profile at 0.2 A/g and (d) long-term galvanostatic cycling at 2 A/g. Structural characterization through ex situ XRD at various charge/discharge states for (e) sat. ZnCl_2_ and (f) 0.6GuaAc/1.9H_2_O.

The performance of the 0.6GuaAc/1.9H_2_O electrolyte was evaluated in a full Zn∥ZnVO cell and benchmarked vs the corresponding sat. ZnCl_2_ cell. The cathode was synthesized following the work by Pang et al.^87^ The XRD analysis (Figure S22a) confirmed the obtained structure of a hydrated form close to ZnV_8_O_23_(H_2_O)_4_ (PDF 04-012-3619), hereafter referred to as ZnVO. The SEM images display elongated clustered rods (Figure S22b). The energy dispersive X-ray (EDX) results indicate a homogeneous distribution of the elements Zn, V, and O, respectively (Figure S22c,d). First, the redox activity of ZnVO was evaluated by using both electrolytes and cyclic voltammetry (CV) (Figure 4a). The CV of the 0.6GuaAc/1.9 H_2_O electrolyte displays two distinct redox peaks, at 0.72/0.52 V and at 0.83/1.00 V, suggesting an enhanced multistep intercalation/deintercalation process attributed to the V^3+^/V^4+^ and V^4+^/V^5+^ redox couples, respectively.^88^ In contrast, for the sat. ZnCl_2_ cell the redox peaks were barely noticeable at 0.5 mV/s and completely absent at 0.1 mV/s (Figure S23a,b). ZnVO cycled with 0.6GuaAc/1.9H_2_O showed no signs of degradation even after 200 cycles (Figure S23c,d) and a good rate capability with 90–99% Coulombic efficiency and effective capacity recovery (Figure 4b). In opposition to the GuaAc electrolyte, the cell with sat. ZnCl_2_ led to unstable cycling and low capacities or even failure (Figure S24). The potential profiles (Figure 4c) confirm the CV results, with two and one pseudo-plateaus for 0.6GuaAc/1.9H_2_O and sat. ZnCl_2_, respectively. Finally, the long-term cycling (Figure 4d, Figure S25) further supports the compatibility of 0.6GuaAc/1.9H_2_O with the ZnVO cathode with a capacity of 190 mAh/g, achieving ca. 1000 cycles and approximately 98% Coulombic efficiency, while the sat. ZnCl_2_ reached a mere <50 mAh/g and failed at 200 cycles.

To further understand the different behaviors, ex situ XRD was performed to analyze any ZnVO structural changes at various charge states (Figure 4e,f). Using the sat. ZnCl_2_ electrolyte, the peak at 7.6° (2θ), which corresponds to the distance between the VO bilayers, shifts to 10° during the first discharge, indicating deintercalation or phase change. Additionally, a new peak at 11.3° emerges during the charge state, suggesting proton intercalation due to an abundance and excess of H^+^ (pH < 0).^89^ Finally, the initial peak at 7.6° was not restored upon charge, suggesting an irreversible process. On the other hand, for the 0.6GuaAc/1.9H_2_O electrolyte, there is a reversible peak shift at 7.6°. First, there is a shift to higher diffraction angles, which indicates a compression of the lattice,^90^ which can be attributed to strong electrostatic interactions between the intercalated Zn^2+^ ions and the VO bilayers.^87,90,91^ The discharge reverses this, indicating stable deintercalation of Zn^2+^. Additionally, no new peaks are detected.

## 3 Conclusions

The optimal ratio of GuaAc to ZnCl_2_ was found to be 0.6:0.4, as it creates an aqueous eutectic mixture being a weakly solvated electrolyte, wherein the Zn^2+^ and Gua^+^ solvation shifted from Zn^2+^–Cl^–^, Zn^2+^–H_2_O, and Gua^+^–Ac interactions to Zn^2+^–Ac, Gua^+^–Cl^–^, and Gua^+^–H_2_O interactions. These specific interactions offer a stable electrolyte over time and a wide temperature range. Additionally, the obtained electrolyte demonstrated a lower viscosity and a higher ionic conductivity, even with high salt concentrations. This is attributed to the chaotropic nature of Gua, which interacts with water molecules while promoting disorder in the overall electrolyte structure. Moreover, the strong kosmotropic nature of Ac further reduces free water and simultaneously solvates Zn ions, leading to partially dehydrated Zn and enhanced Zn stability. Furthermore, we demonstrated the importance of fine-tuning the water content in the Zn aqueous electrolyte. Adjusting the water content altered the local structure, especially in the diluted 0.6GuaAc/2.2H_2_O electrolyte, as indicated by DSC measurements. The reported electrolyte with optimized water content shows enhanced stability of the Zn anode and vanadium-based cathode in comparison to sat. ZnCl_2_. Hence, by selection of specific and adequate ions, the Zn solvation and electrolyte structure can be tuned to obtain an efficient Zn electrolyte. This effective and simple strategy can be applied to other aqueous electrolytes to develop more efficient and stable aqueous batteries.

## 4 Methods

### 4.1 Materials

Ultradry zinc chloride (ZnCl_2_) pearls (99.99%) were purchased from Sigma-Aldrich and kept inside a glovebox (0.5 ppm O_2_, 0.1 ppm H_2_O, argon gas) for preparation of the electrolyte. Guanidinium acetate (GuaAc) (≥99%) and vanadium pentoxide (V_2_O_5_) (≥99.5%) were purchased from Sigma-Aldrich. Zinc chloride powder (≥98%) and *N*-methyl-2-pyrrolidone (NMP) (99.5%) were acquired from Alfa Aesar for preparation of the cathode material. Super P carbon black (>99%) was provided by Thermo Scientific. Polyvinylidene fluoride (PVDF) (≥99.5%) and copper foil (99.95%, 10 μm) were purchased from MTI. Zinc foil (99.95%, 100 μm) was purchased from Goodfellow. Alumina polishing solution from ALS-Japan (0.05 μm) was used to clean the zinc foil, followed by sonicating for 5 min in acetone. Carbon paper (200 μm) was provided by Caplinq. Ultrapure water (18 μS/cm) was used in all experiments.

### 4.2 Electrolyte Preparation

The (GuaAc)_*x*_(ZnCl_2_)_1–*x*_/*n*H_2_O electrolytes were obtained by mixing the corresponding salt molar ratio *x*, followed by adding water in small increments while stirring on a hot plate set to 30 °C until complete dissolution was achieved. The obtained electrolytes were left stirring for 2 h at room temperature and then overnight without stirring to ensure that the salts remained dissolved before conducting any experiments. The ultradry ZnCl_2_ was weighed inside the glovebox to avoid any water uptake from the atmosphere. The weighing of GuaAc was done outside the glovebox. The solutions were prepared according to molality, i.e., moles of solute per kilogram of solvent (water). Saturated ZnCl_2_ and saturated GuaAc were prepared using the same procedure. The different water contents of the 0.6GuaAc electrolytes were obtained by adding water while stirring at 30 °C until the desired salt/water ratios were obtained.

### 4.3 ZnVO Cathode Synthesis and Preparation

The synthesis of ZnVO powder used a modified protocol of the method proposed by Pang et al.^92^ First, 1.5 g of V_2_O_5_ was added to a solution of 2 g of ZnCl_2_ powder dissolved in 22.5 mL of water. The slurry was left stirring for 128 h at 50 °C. The obtained red product was washed with water and ethanol, 5 times each. The rinsed product was then dried at 60 °C without vacuum for 24 h before it was ground to a powder for physicochemical and electrochemical characterization. The theoretical capacity of ZnVO is 323 mAh/g.^93^

The obtained ZnVO powder was mixed with Super P carbon black and PVDF in a mass ratio of 7:2:1. NMP was slowly added while stirring until a slurry of desired consistency was acquired. The slurry was stirred overnight before being cast on carbon paper with a wet thickness of 150 μm. The carbon paper with the ZnVO slurry was left to dry overnight in an oven at 60 °C without vacuum before being manually punched into disk-shaped cathodes (Ø 8 mm, loading 3.1–3.8 mg/cm^2^).

## Supporting Information Available

Characterization, DFT calculation, and electrochemical measurement details; photographs of prepared electrolytes; DSC measurements; Raman spectra combined with DFT-calculated vibrational frequencies; experimental and computed PDF spectra with calculated interatomic distances; electrolyte transport properties evaluated with VFT-fitted data and plots; Zn^2+^ transference number; ESW figures and calculated modified Aurbach Coulombic efficiency with corresponding potential profiles; potential profiles of the conducted rate capability and long-term cycling experiments of the Zn anode; SEM images of the recovered Zn anode; XPS results; impedance spectra with the fitted circuit; characterization of the synthesized ZnVO material; and electrochemical data of Zn∥ZnVO cells (PDF)
