# Supplementary material for: Tailoring the Solvation of Aqueous Zinc Electrolytes by Balancing Kosmotropic and Chaotropic Ions
Source: ACS Nano. 2025 Feb 6;19(6):6388–98. doi: 10.1021/acsnano.4c16521 (PMC11841044; doi:10.1021/acsnano.4c16521)
Supplement: Supplementary file 1 — nn4c16521_si_001.pdf [file nn4c16521_si_001.pdf]

Electronic Supplementary Information (ESI)

Supporting Information

Tailoring the Solvation of Aqueous Zinc Electrolytes by Balancing Kosmotropic and Chaotropic Ions

Ibrahim Al Kathemi[a], Zaher Slim[b], Fernando Igoa Saldaña[c], Ann-Christin Dippel[c], Patrik Johansson[b,d], Mateusz Odziomek[a] and Roza Bouchal[a*]

[a]*Department of Colloid Chemistry, Max Planck Institute of Colloids and Interfaces, Am Mühlenberg 1, 14476 Potsdam, Germany.*

[b]*Department of Physics, Chalmers University of Technology, 41296 Gothenburg, Sweden.*

[c]*Deutsches Elektronen-Synchrotron DESY, Notkestraße 85, 22607 Hamburg, Germany.*

[d]*Alistore-European Research Institute, CNRS FR 3104, Hub de l'Energie, Rue Baudelocque, 80039 Amiens, France.*

**roza.bouchal@mpikg.mpg.de*

Contents

Characterizations 3
Differential scanning calorimetry 3
Ionic conductivity 3
Viscosity 3
Vogel-Fulcher-Tammann and Walden model 3
Raman spectroscopy 4
X-ray total scattering 4
X-ray diffraction 4
X-ray photoelectron spectroscopy 4
Scanning electron microscopy and energy-dispersive x-ray spectroscopy 4
Density functional theory calculations 4
Electrochemical measurements 5
Transference number 5
Linear sweep voltammetry 5
Modified Aurbach coulombic efficiency 5
Desolvation energy 5
Zn||Zn stability 6
Zn||ZnVO full cell stability 6
Supporting information figures S1-25 7
Supporting information tables S1-9 26
References 33

Characterizations

Differential scanning calorimetry

DSC was performed on a DSC 204 F1 Phoenix (Netzsch, Selb, Germany) using an aluminum pan with a pierced lid with 10 ± 1 mg of sample, in a temperature range of +50 to -150 °C. The cooling and heating rate were both 10 K/min with a constant temperature time set to 2 minutes.

Ionic conductivity

The ionic conductivity of all the electrolytes was determined by electrochemical impedance spectroscopy. Two electrode cells were calibrated and the cell constant k_C was determined accordingly. The k_C were in the range of 0.9 – 1.1. The frequency sweep was from 100 kHz to 50 mHz with a potential amplitude of 10 mV. The measurements were conducted in the temperature range of -20 to 80 °C with a stabilization time of 1 hour at each temperature. The ionic resistances were determined as the curve intercepts with the real part of the impedance axis.

Viscosity

The viscosity was measured with an Automated Microviscometer (AMVn, Anton Paar, Graz) which utilizes the Hoeppler's falling ball principle. A capillary with an internal Ø 1.6 mm was filled with a solution and a stainless-steel ball with Ø 1.5 mm was placed inside. The capillary was kept at an angle of 70° for 6 repeated measurements done in duplicates in the temperature range of 10 to 70 °C. For sat. $ZnCl_2$ electrolyte, a gold ball of 3 mm and a capillary with an internal Ø 4 mm was used to avoid potential corrosion of the device. The density was determined in a density oscillation tube (DMA 5000M, Anton Paar, Graz) in the same temperature range as the viscosity.

Vogel-Fulcher-Tammann and Walden model

The Vogel–Fulcher–Tammann equation is used to describe the viscosity of liquids as a function of temperature, particularly emphasizing its strong temperature-dependent variation in the supercooled regime as it approaches the glass transition. The model is described by the following formula:

$$(S1)\ B = B_0^i * \exp\left(\frac{-B^i}{T - T_0^i}\right)$$

where B_o^i is a pre-exponential term which corresponds to either the ionic conductivity (σ_0) or viscosity (η_0) at infinite temperature. B^i is a pseudo-activation energy and corresponds to the slope of the VFT plots. B^i can be correlated to the activation energy barrier E_a^i through $B^i = E_a^i$ / R with R being the gas constant. The parameter T_o^i is the dynamic glass transition temperature and correlates to the T_g measured in DSC. The fitted parameters are summarized in Tables S7 and S8.

The Walden plot can be utilized to better understand the relationship between ionic conductivity and viscosity, as well as to evaluate the ionicity of each electrolyte.[1], [2] The Walden rule is given by:

$$(S2)\ Log(\Lambda) = Log(C) + \alpha * \log(\eta^{-1})$$

Where Λ is the molar conductivity ($\Lambda = \sigma * M/\rho$) in $S{\cdot}cm^2/mol$, M is the molar weight, C is a constant, α is an experimental value between 1 and 0 and η is the viscosity. If α reaches a value of 1, there is a total ionic dissociation in the electrolyte which is given by the ideal 0.01 M KCl line. To obtain the Walden plot, a correction was made as described by Yang et al.[1].

Raman spectroscopy

The Raman spectra were obtained on a WITec Alpha 300M+ instrument and collected in a backscattering geometry in a confocal mode with a 20x objective. Each measurement used a 532 nm laser at a power of 50 mW, an integration time of 0.5 seconds and 60 accumulations with a resolution of 2 cm^{-1}. A quartz cuvette with a thickness of 2 mm was filled with the electrolyte. The obtained spectra were fitted with either Lorentz, Gaussian or Gaussian+Lorentz (Voigt) functions.

X-ray total scattering

X-ray scattering was acquired at the PETRA III synchrotron at Deutsches Elektronen-Synchrotron (DESY) at the beamline P21.1 with an X-ray energy of 101.4 keV. A PerkinElmer XRD detector was used at a distance of 0.4 m from the sample. The data ranged from q = 0.1 to 24 $Å^{-1}$, corresponding to length scales of 1 to 30 Å. The sample-to-detector distance was calibrated using a Ni standard. A glass capillary with a wall thickness of 0.01 mm of each sample was prepared. The data processing and analysis were performed using pyFAI[3] and xPDFSuite[4] to integrate the 2D data to one dimensional patterns and subsequently extract the pair distribution functions (PDF) in real space[3]. An empty capillary was used as a background. All attained patterns were normalized to their relative maxima.

X-ray diffraction

X-ray Diffraction (XRD) measurements were performed on a Rigaku SmartLab diffractometer. The apparatus uses Cu Kα radiation at 1.5406 Å. The samples were analyzed with steps of 0.05° and a scanning rate of 1°/min.

X-ray photoelectron spectroscopy

XPS measurements were performed using a Thermo Fisher Scientific K-Alpha, and data were treated with CasaXPS software. The Zn foils were pressed on carbon tape deposited on a sample holder and analyzed through monochromatic X-ray radiation of 1486.6 eV (Al K*α).* Survey scans were obtained using a pass energy of 200 eV and step size of 1 eV, with a spot size of approximately 100 µm. High-resolution spectra of Zn 2p (1015–1052 eV), C 1s (280–298 eV), O 1s (526–545 eV), Cl 2p (196–203 eV) and N 1s (394–404 eV) were recorded using a pass energy of 50 eV and step size of 0.1 eV. The binding energy was calibrated against the 284.8 eV peak of adventitious carbon (C–C)[5]. A U2 Tougaard background was used and peaks were deconvoluted using mixed Gauss-Lorentz sum function line shape GL(30). The electron kinetic energies of the different regions are between 480 eV and 1280 eV, typically corresponding to electron inelastic mean free paths (IMFP) *ca.* 0.7 nm and 1.9 nm, leading to a probed depth between 2.1 nm and 5.7 nm (3 x IMFP)[6]. Relative Sensitivity Factors (RSF) of 1, 1.8, 2.93, 28.72, and 2.285 were used for C, N, O, Zn, and Cl, respectively.

Scanning electron microscopy and energy-dispersive x-ray spectroscopy

Scanning electron miscroscope (SEM) and energy-dispersive x-ray spectroscopy (EDX) were performed on a LEO 1550 Gemini Zeiss microscope operating at 5 kV and 10 kV, respectively. The samples were only dried under vacuum for 10 minutes before transferring them to the SEM.

Density functional theory calculations

Density functional theory (DFT) calculations were carried out using Gaussian 16[7]. All structures were manually drawn in the Gaussview software and fully optimized in both the gas and the solvent phase. All results were obtained using Becke's three parameter exchange correlation functional (B3PW91)[8]

together with the 6311+G(d) basis set[9]. Water was used as the solvent for all calculations using a continuum solvation model based on the self-consistent reaction field approach. Raman frequencies were calculated and all structures were found to be a local minima on the potential energy surfaces as confirmed by the absence of any imaginary frequencies.

Electrochemical measurements

Transference number

The transference number of Zn^{2+} ($t_{Zn}{}^{2+}$) was determined using a two-compartment Swagelok cell with the WE and CEs both being a Ø 10 mm Zn foil. Two glass fiber separators and 150 µl electrolyte was used. The Swagelok cells were left to rest overnight before conducting an experiment. The $t_{Zn}{}^{2+}$ was calculated using the Evans-Vincent-Bruce equation[10], [11]:

$$(S3)\ t_{Zn^{2+}} = \frac{I_s * (\Delta V - I_0 * R_0)}{I_0 * (\Delta V - I_s * R_s)}$$

With ΔV being the potential size, I_0 and R_0 the current and resistance at initial state, and I_s and I_R the stable current and reaction resistance after polarization, respectively. The ΔV was set to 10 mV and the frequency rate of the PEIS was 100 kHz to 100 mHz. All experiments were performed in triplicates.

Linear sweep voltammetry

Linear sweep voltammetry was conducted at a scan rate of 0.2 mV/s using a three-compartment vial with Ø 1.2 mm glassy carbon as the working electrode (WE), platinum wire as the counter electrode (CE) and Ag/AgCl reference electrode (RE, 3 M NaCl). Each scan started from the open circuit voltage and then scanned towards reducing or oxidating potentials. The electrodes were submerged in 2 ml electrolyte and degassed for 2 minutes under nitrogen gas. For each experiment, new electrolyte was used.

Modified Aurbach coulombic efficiency

The Coulombic efficiency was determined through a modified Auerbach method proposed by Vazquez et al[12]. The protocol uses a two-compartment Swagelok cell with a Ø 12 mm Cu foil WE, a Ø 10 mm Zn foil as CE, a Ø 13 mm glass fiber separator and 90 µl of electrolyte. The experimental method consists of three sequences. First, excess amount of Zn was plated, stripped and re-plated with a current density of 3 mA/cm^2 and specific areal capacity of 5 mAh/cm^2. Secondly, Zn plating and stripping was performed with 0.5 mA/cm^2 and 1 mAh/cm^2 for 10 charge-discharge cycles. Finally, Zn stripping was carried out with a current density of 3 mA/cm^2 and a cut off voltage of 0.5 V vs. Zn/Zn^{2+}.

Desolvation energy

The desolvation energy (E_{des}) was determined using a two-compartment Swagelok cells with Ø 10 mm Zn electrodes, two Ø 13 mm glass fiber separator and 150 µl of electrolyte in the temperature range of 20 to 60 ^oC. The cell was first stabilized for 10 hours, followed by 5 initial cycles at 0.5 mA/cm^2 and 0.4 mAh/cm^2. Then, each temperature was set separately and after reaching a stable temperature, the cell was left for 1 hour extra at that temperature before conducting electrochemical impedance measurements from 100 kHz to 50 mHz wth a voltage amplitude of 10 mV.

Zn||Zn stability

Cycling stability of Zn||Zn cells were conducted in two-compartment Swagelok cells with Ø 10 mm Zn electrodes, a Ø 13 mm glass fiber separator and 90 µl of electrolyte. After resting the cells for 10 hours, a rate capability consisting of 10 cycles of 0.2, 0.5, 1, 2 and 3 mA/cm^2 with 0.4 mAh/cm^2 each was performed. Following this, 0.5 mA/cm^2 with the same capacity was applied as a long-term cycle stability test. To compare SEM images, new Swagelok cells were made and tested under 1 mA/cm^2 for 40 hours before dissembling the cell before conducting SEM. The cut off potential was 0.5 V.

Zn||ZnVO full cell stability

Full cell experiments with Zn||ZnVO were performed in a two-electrode Swagelok cell using ZnVO with Ø 8 mm as WE and Zn foil with Ø 10 mm as CE. 90 µL of electrolyte was used to wet the glass fiber separator (Ø 13 mm). The rate capability test was conducted by applying varying current rates from 0.1 A/g to 5 A/g, and finally returning to 0.1 A/g. The long term cyclability was performed under an applied current rate of 2 A/g. For the *ex situ* XRD analysis, new cathodes of the ZnVO material were cut for each step. The recovered cathodes were washed with DI water before drying them overnight at 50 ^{o}C without vacuum. All experiments started with a discharge cycle.

Supporting information figures S1-25

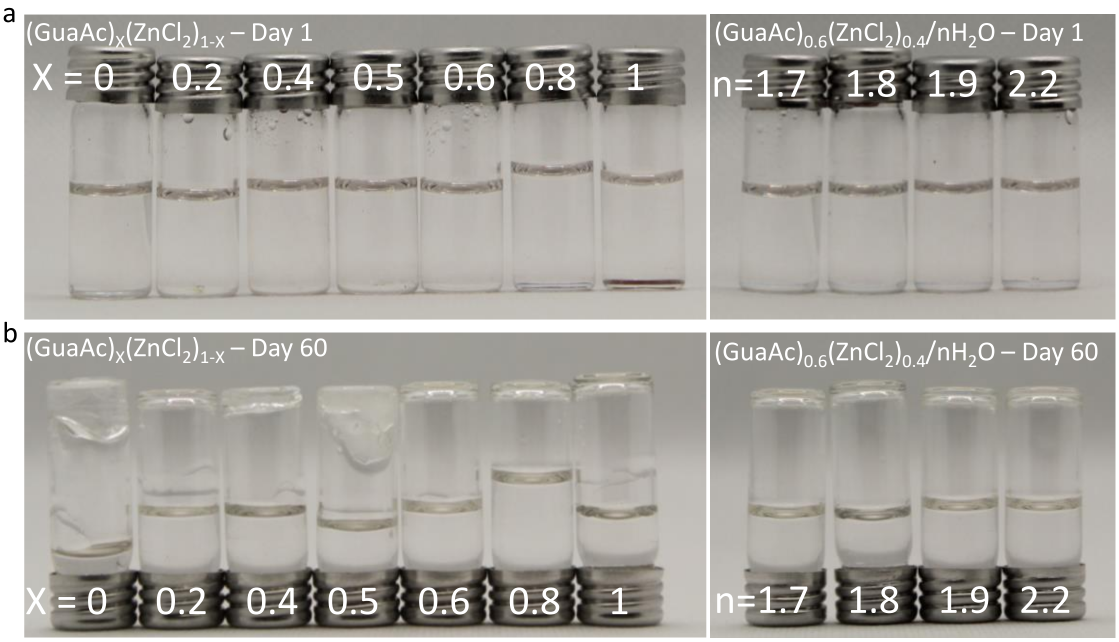

Figure S1: Photographs of the electrolytes with different salt ratios and 0.6GuaAc with different water contents. a) After 1 day. b) After 60 days.

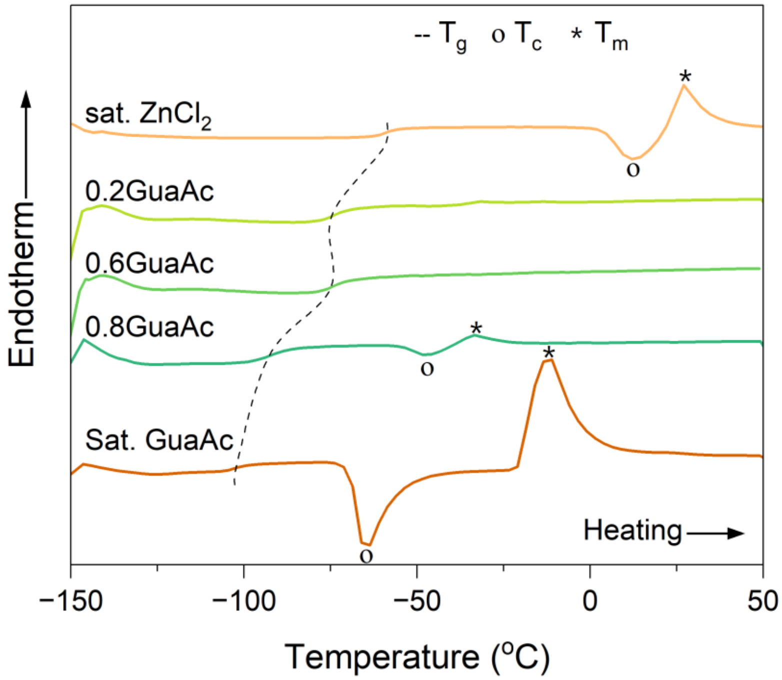

Figure S2: DSC traces of the mixed electrolytes as compared to the sat. ZnCl2 and the sat. GuaAc electrolytes.

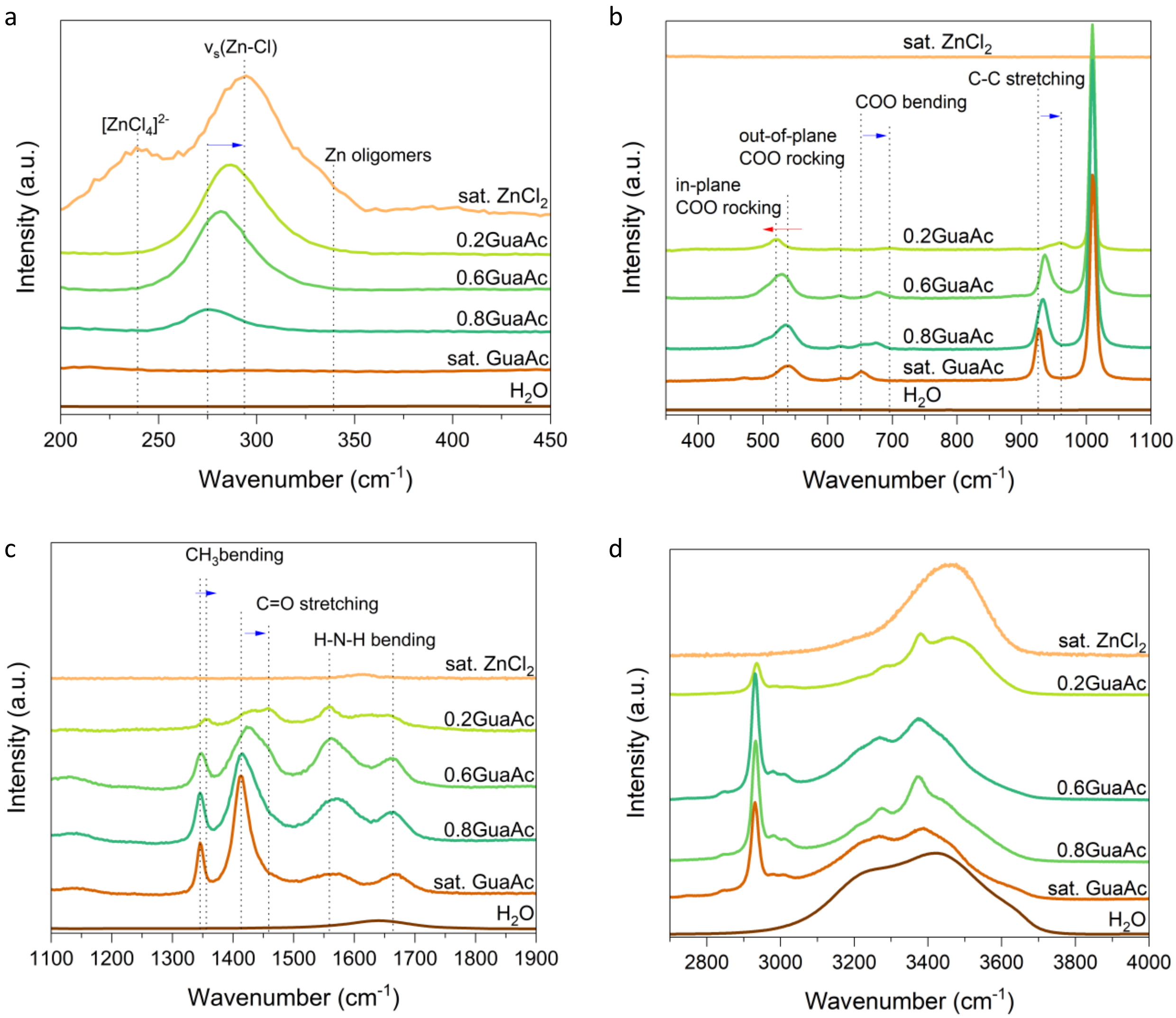

Figure S3: Raman spectra of the mixed GuaAc/$ZnCl_2$ electrolytes as compared to the sat. $ZnCl_2$ and the sat. GuaAc electrolytes. a) Zn^{2+} solvation region (200-450 cm^{-1}). b-c) Acetate anion region (400-1900 cm^{-1}). d) Water OH-stretching region (2800-3800 cm^{-1}). The N-H bond vibrations of Gua are also present in the acetate and water regions.

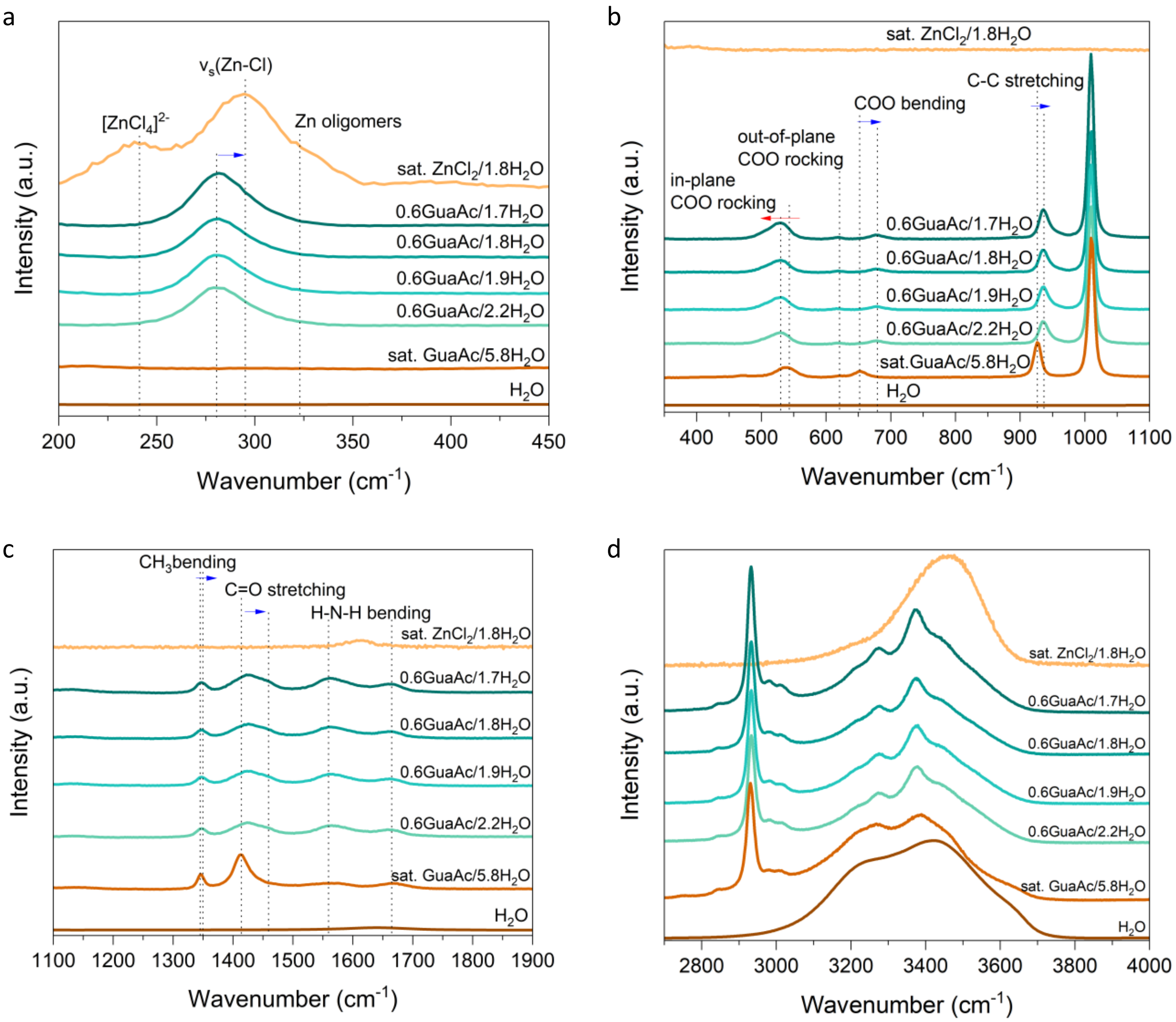

Figure S4: Raman spectra of the mixed $(GuaAc)_{0.6}(ZnCl_2)_{0.4}/nH_2O$ electrolytes as compared to the sat. $ZnCl_2$ and the sat. GuaAc electrolytes. a) Zn^{2+} solvation region (200-450 cm^{-1}). b-c) Acetate anion region (400-1900 cm^{-1}). d) Water OH-stretching region (2800-3800 cm^{-1}). The N-H bond vibrations of Gua are also present in the acetate and water regions.

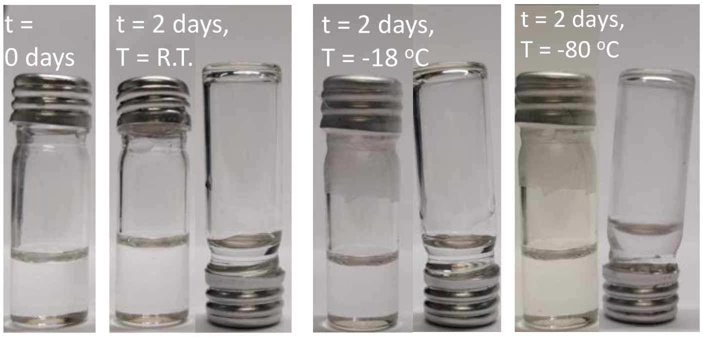

Figure S5: Stability of $(Zn(Ac)_2)_{0.33}(GuaCl)_{0.67}/1.6H_2O$ electrolyte (reaction (3)) at room temperature (R.T.), -18 °C and -80 °C.

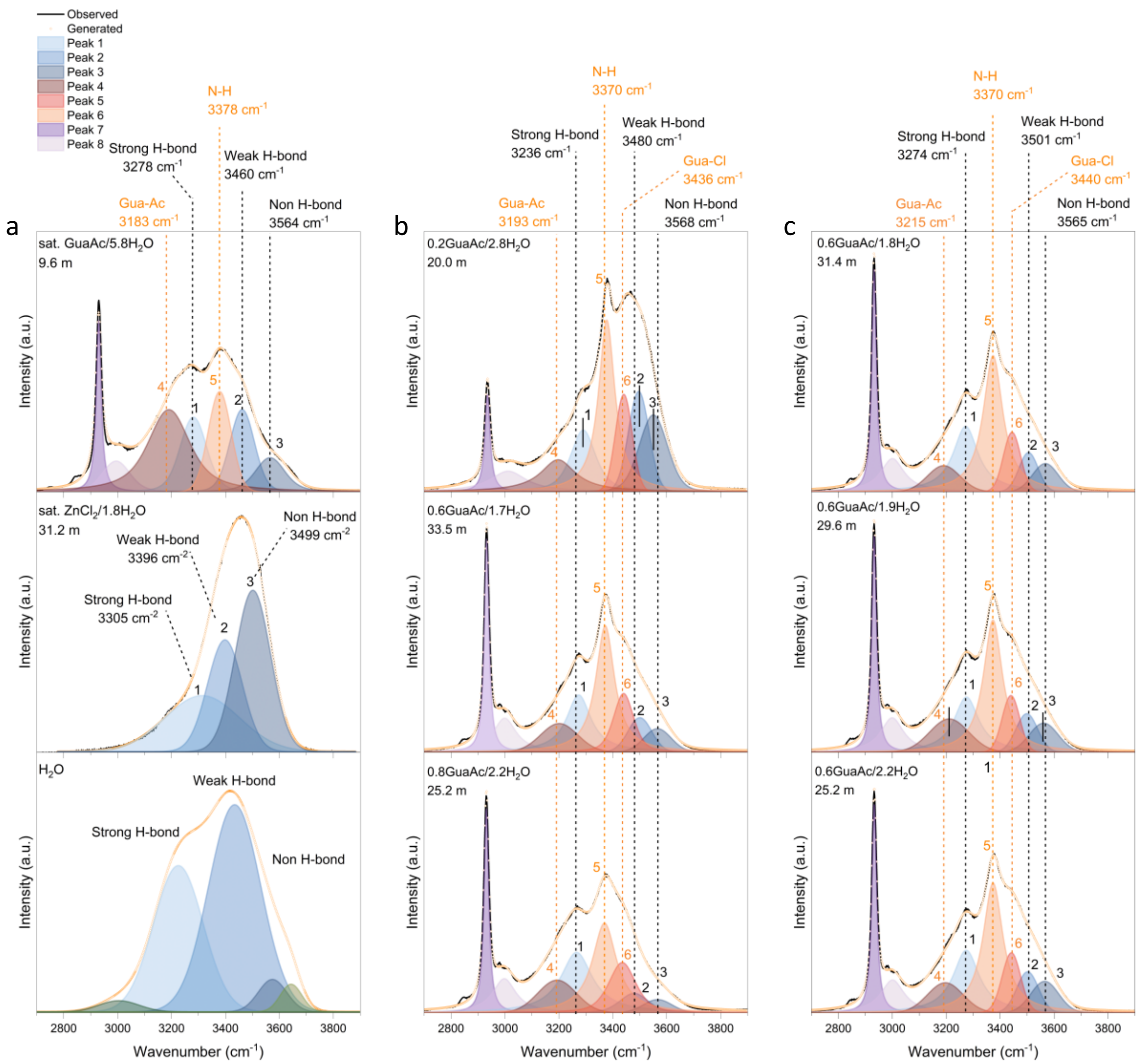

Figure S6: Deconvolution of the water region in the Raman spectra. a) Comparison of sat. GuaAc, sat. $ZnCl_2$ and ultra-pure H_2O. b) Comparison of 0.2, 0.6 and 0.8GuaAc. c) Comparison of 0.6GuaAc with 1.8, 1.9 and 2.2 salt/water ratio.

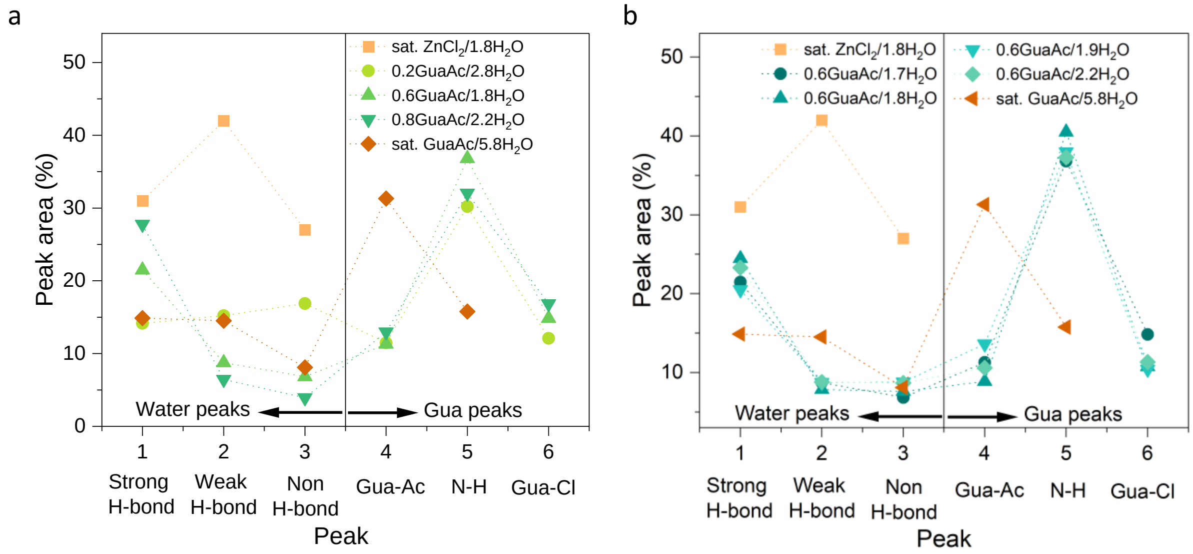

Figure S7: Comparison of the peak area percentage of the water/N-H region (3000 – 3800 cm^{-1}). a) comparison of the mixed electrolytes with sat. $ZnCl_2$ and sat. GuaAc. b) comparison of the 0.6GuaAc electrolytes with sat. $ZnCl_2$ and sat. GuaAc.

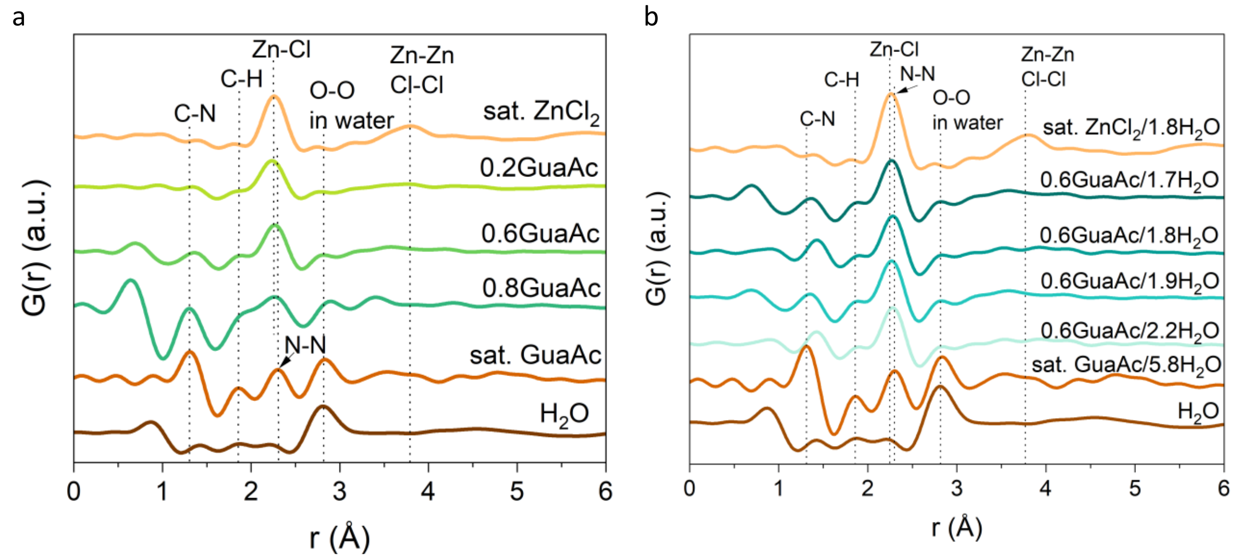

Figure S8: PDF of GuaAc/ZnCl₂ based electrolytes as compared to sat. ZnCl2 and sat. GuaAc. a) Different salt ratios of GuaAc/ZnCl₂ electrolytes. b) 0.6GuaAc electrolytes with varying water content.

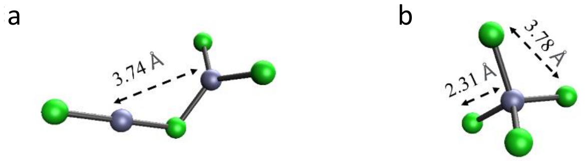

Figure S9: DFT calculated interatomic distances for a) structure (i) and b) structure (iii).

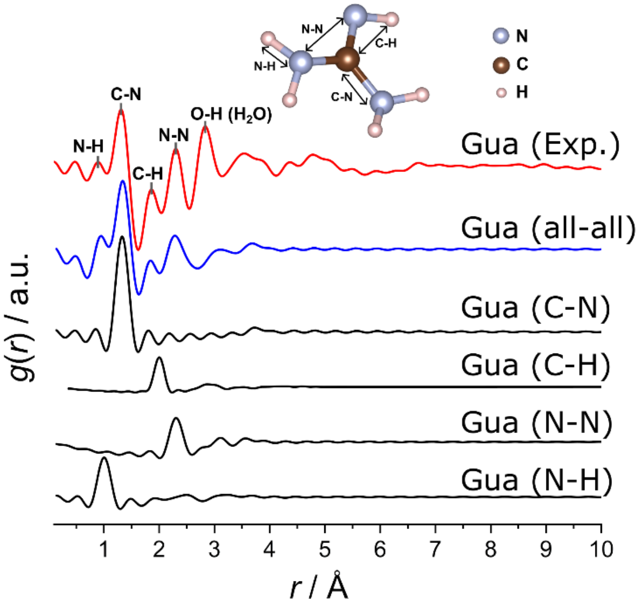

Figure S10: Computed partial spectra of different bond lengths of Gua.

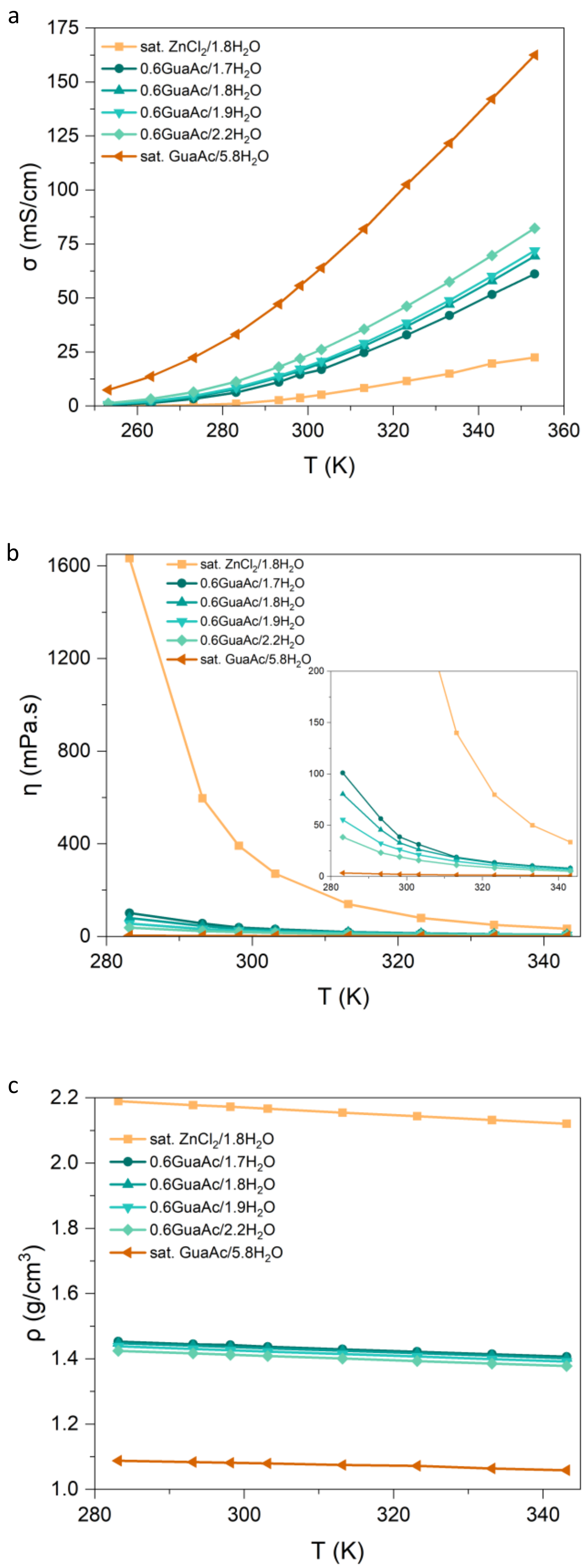

Figure S11: Physico-chemical properties of a few selected electrolytes. a) Ion conductivity in the temperature range of -20 to 80 °C. b) Viscosity and c) density in the temperature range of 10 to 70 °C.

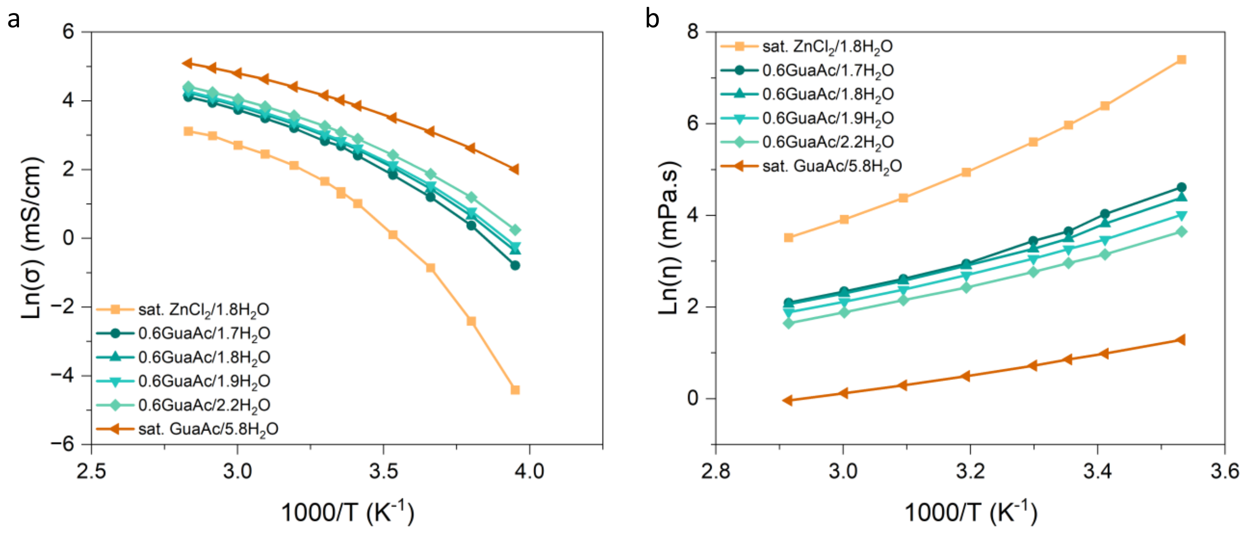

Figure S12: Arrhenius plots. a) Variation of the natural log of the ion conductivity in the temperature range of -20 to 80 °C. b) Variation of the natural log of the viscosity in the temperature range of 10 to 70 °C.

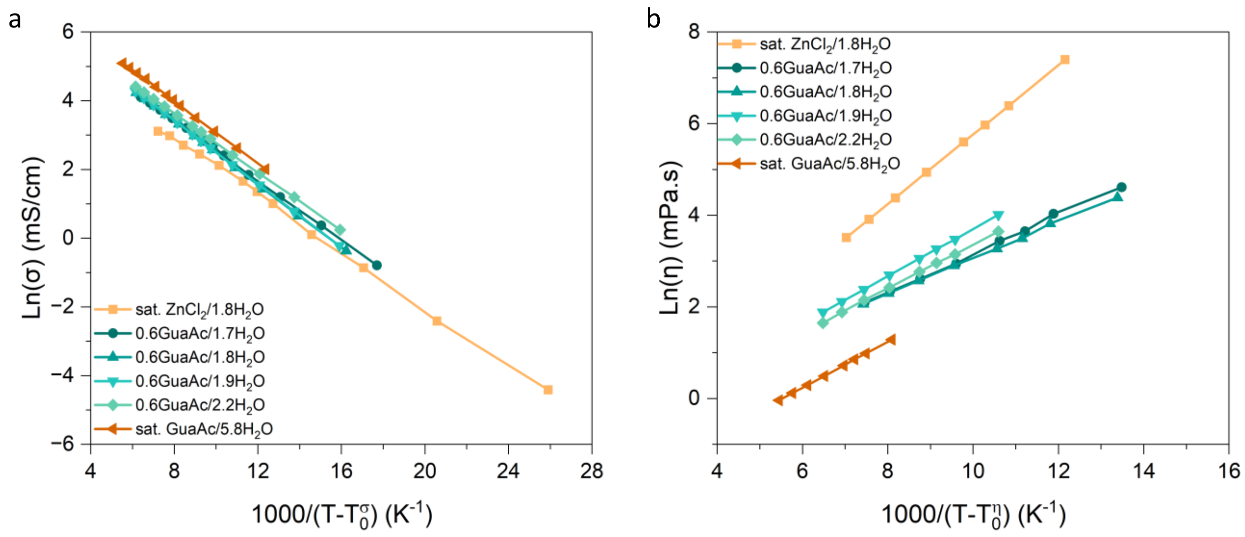

Figure S13: VFT plots. a) Variation of the natural log of the ion conductivity in the temperature range of -20 to 80 °C. b) Variation of the natural log of the viscosity in the temperature range of 10 to 70 °C.

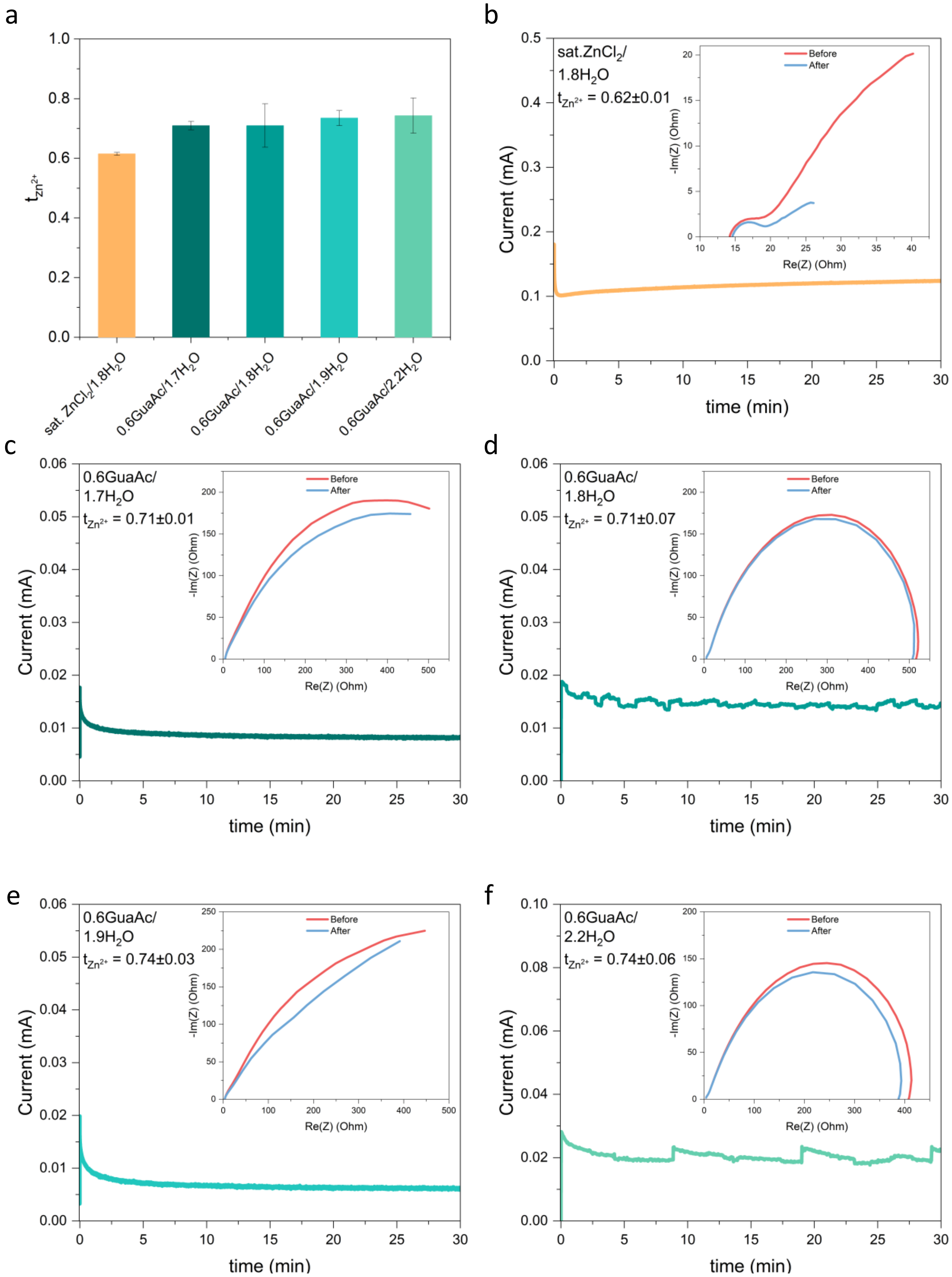

Figure S14: Determination of the Zn^{2+} transference number at a 10 mV overpotential in symmetric Zn||Zn cells. a) The determined Zn^{2+} transference number of each electrolyte. b-f) Chrono-amperograms of sat. $ZnCl_2$ and the selected 0.6GuaAc electrolytes.

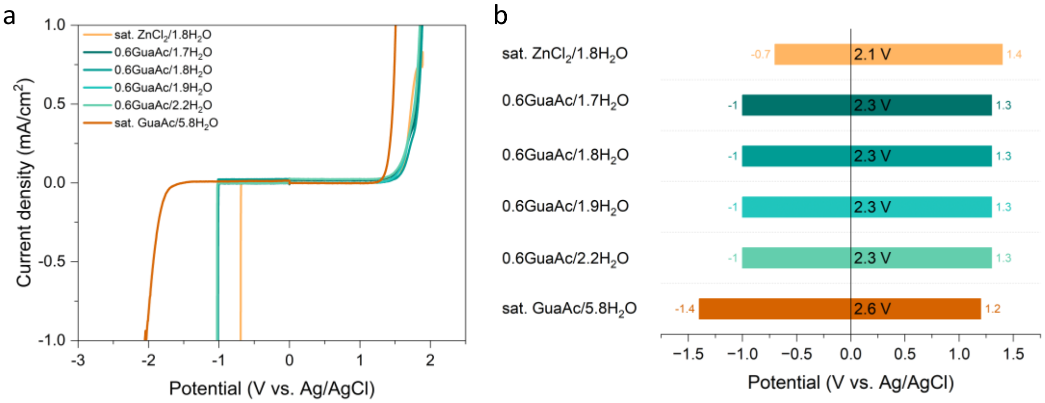

Figure S15: ESW of the 0.6GuaAc electrolytes as compared to the sat. $ZnCl_2$ and the sat. GuaAc electrolytes. a) Profiles of the LSV traces. b) Oxidation and reduction limits.

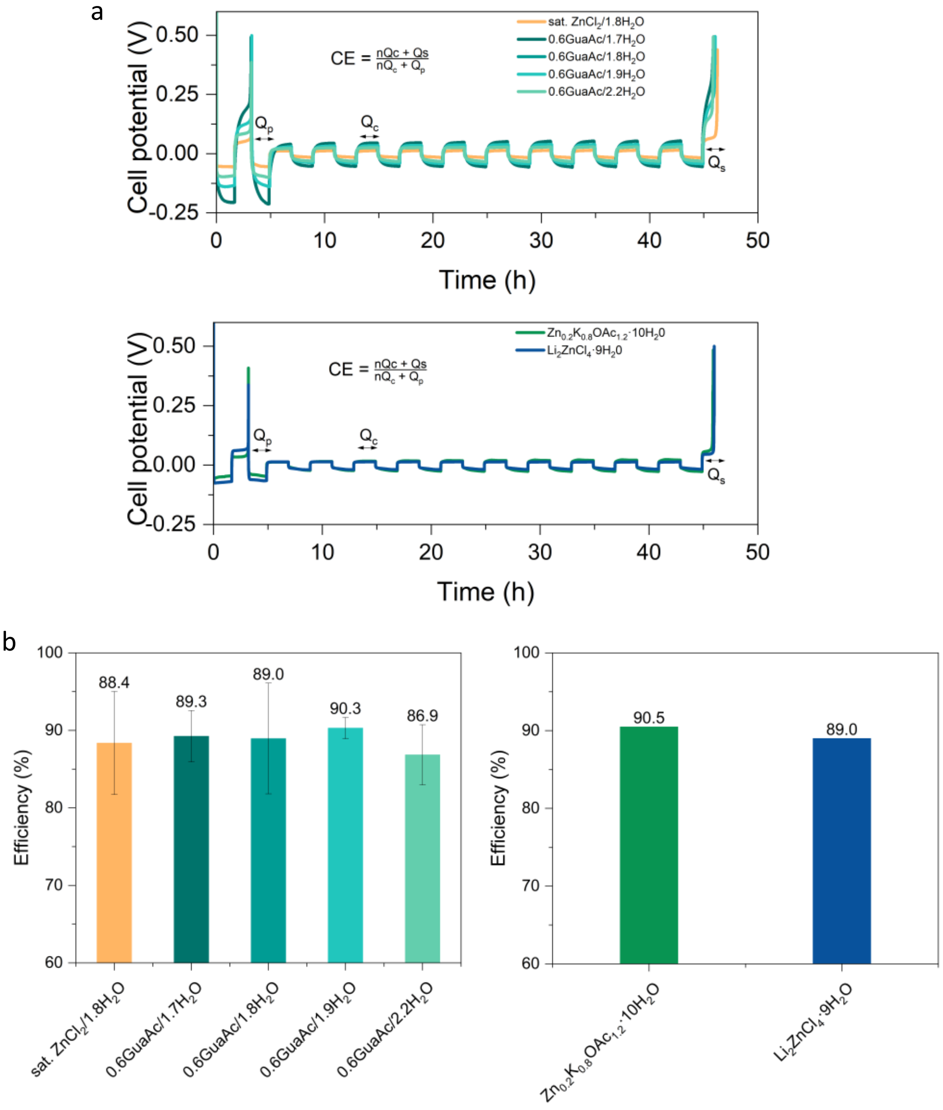

Figure S16: Modified Aurbach coulombic efficiency test for sat. $ZnCl_2$, 0.6GuaAc based and literature electrolytes. a) Potential profile for the sat. $ZnCl_2$, 0.6GuaAc based electrolytes (top) and the literature electrolytes (bottom). b) Calculated Coulombic efficiencies for the sat. $ZnCl_2$ and 0.6GuaAc based (left) and the literature electrolytes (right).

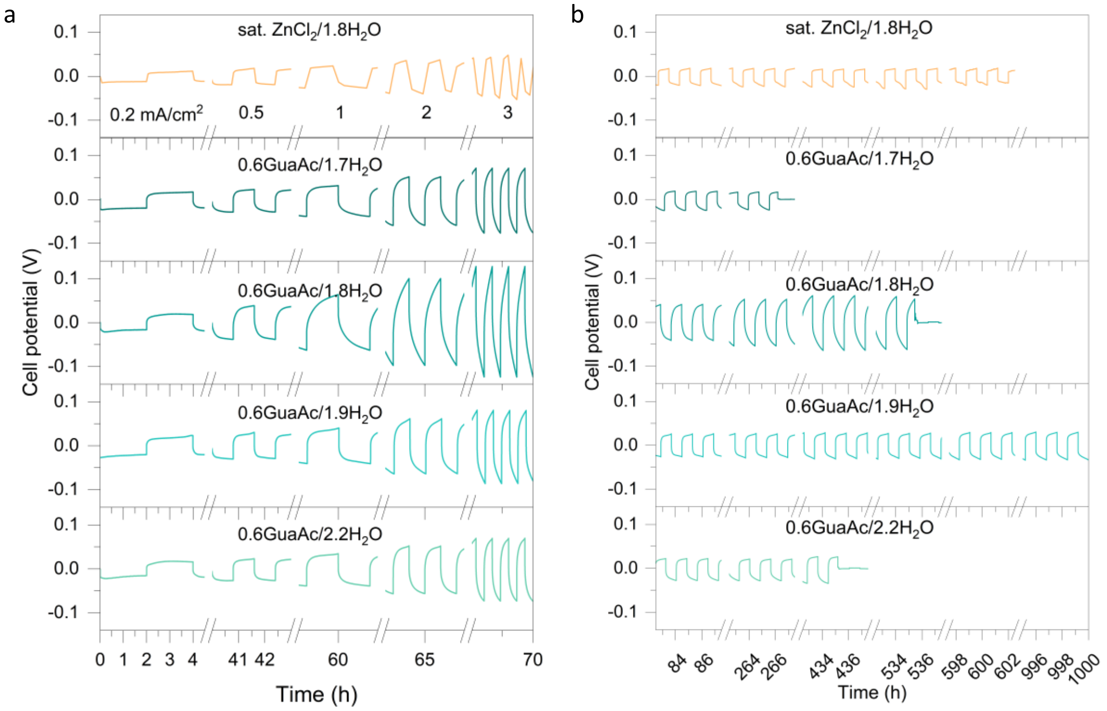

Figure S17: Potential profiles of the selected GuaAc/ZnCl₂ electrolytes with a capacity of 0.4 mAh/cm². a) Rate capability profiles. b) Long term cycling at 0.5 mA/cm².

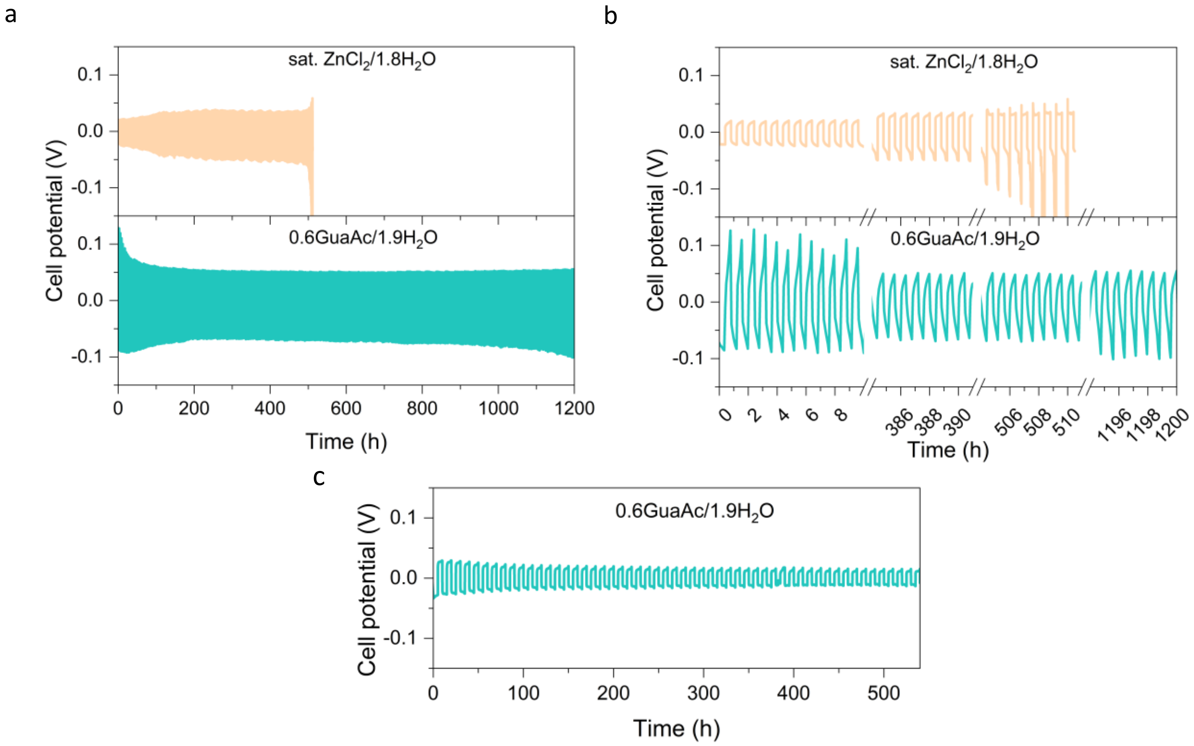

Figure S18: Long-term cycling performance of the Zn anode for 0.6GuaAc/1.9H₂O and sat. ZnCl₂ electrolytes at different applied current and capacity. a-b) Performance and potential profile at 1 mA/cm² and 0.4 mAh/cm². c) Performance at 0.5 mA/cm² and 2.5 mAh/cm².

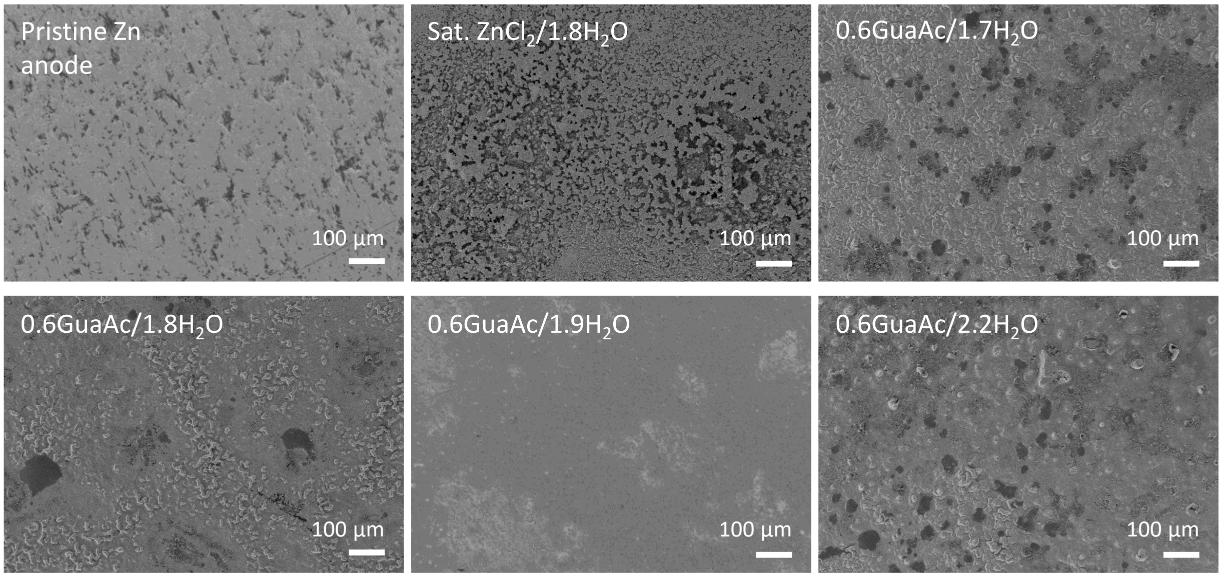

Figure S19: SEM pictures of the recovered Zn anode after 10 cycles of galvanostatic cycling at 0.5 mA/cm^2 and 0.4 mAh/cm^2 for 0.6GuaAc/nH$_2$O and sat. ZnCl$_2$ in a Zn||Zn cell.

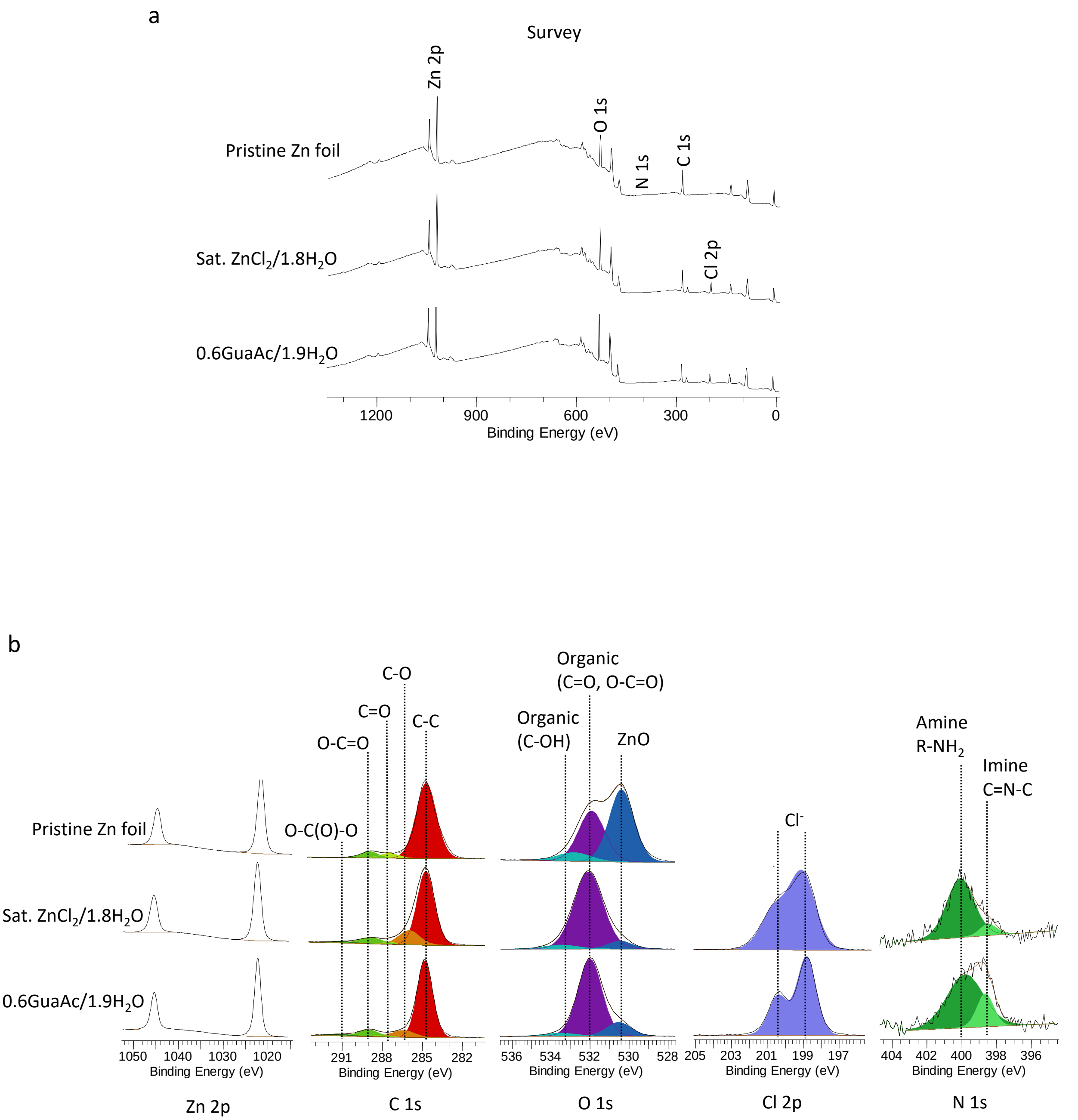

Figure S20: XPS results of the Zn anode after 50 galvanostatic cycles at 1 mA/cm^2 and 0.4 mAh/cm^2 for the selected electrolytes in a Zn||Zn cell. a) Survey. B) Deconvolution of different XPS regions.

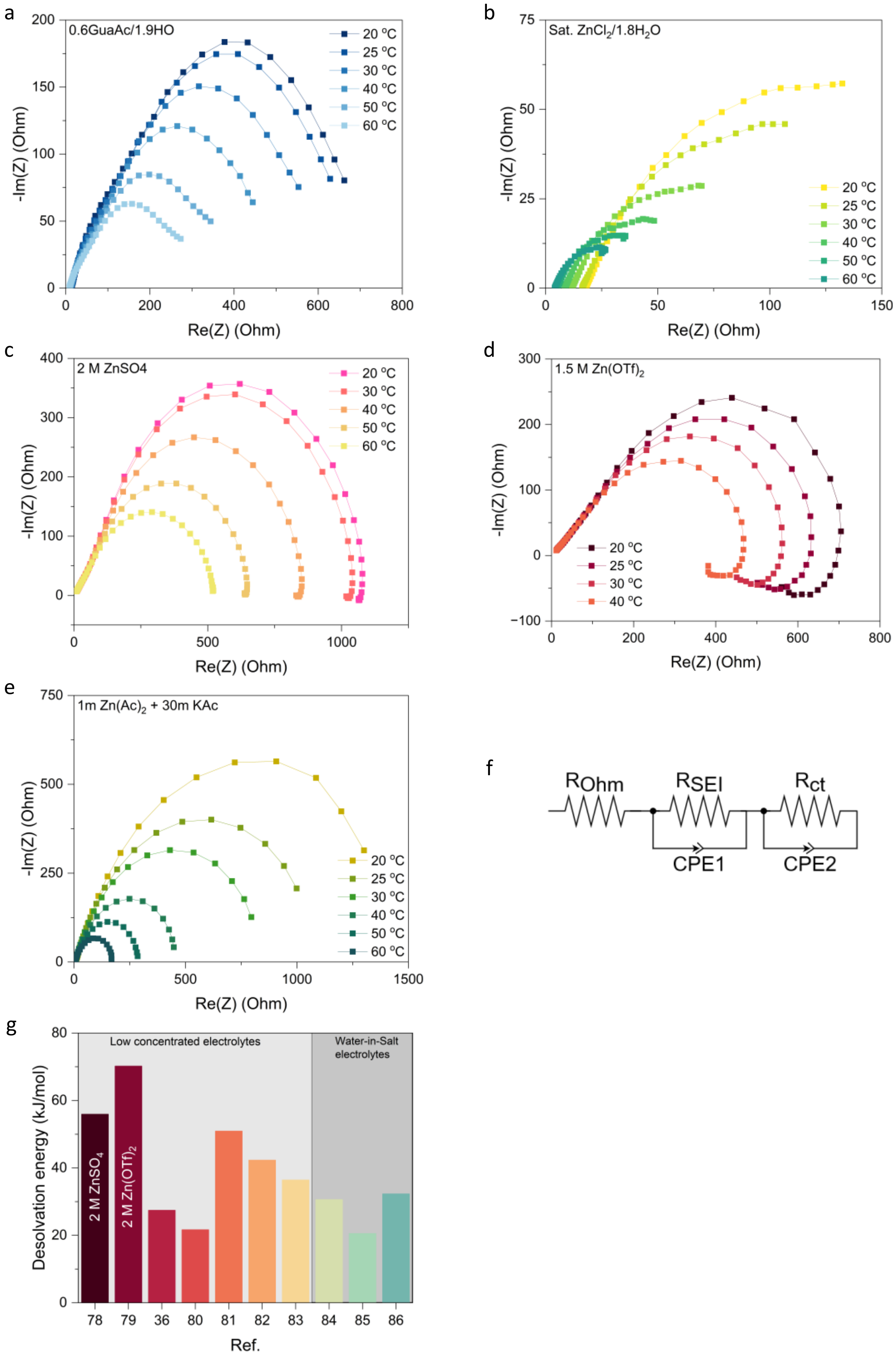

Figure S21: EIS profiles for determining the desolvation energy. a-e) Chosen dilute and concentrated electrolytes. f) Chosen circuit for fitting the data with R_{Ohm} the ohmic resistance, R_{SEI} the SEI layer resistance, R_{ct} the charge transfer resistance and CPE the constant phase element.

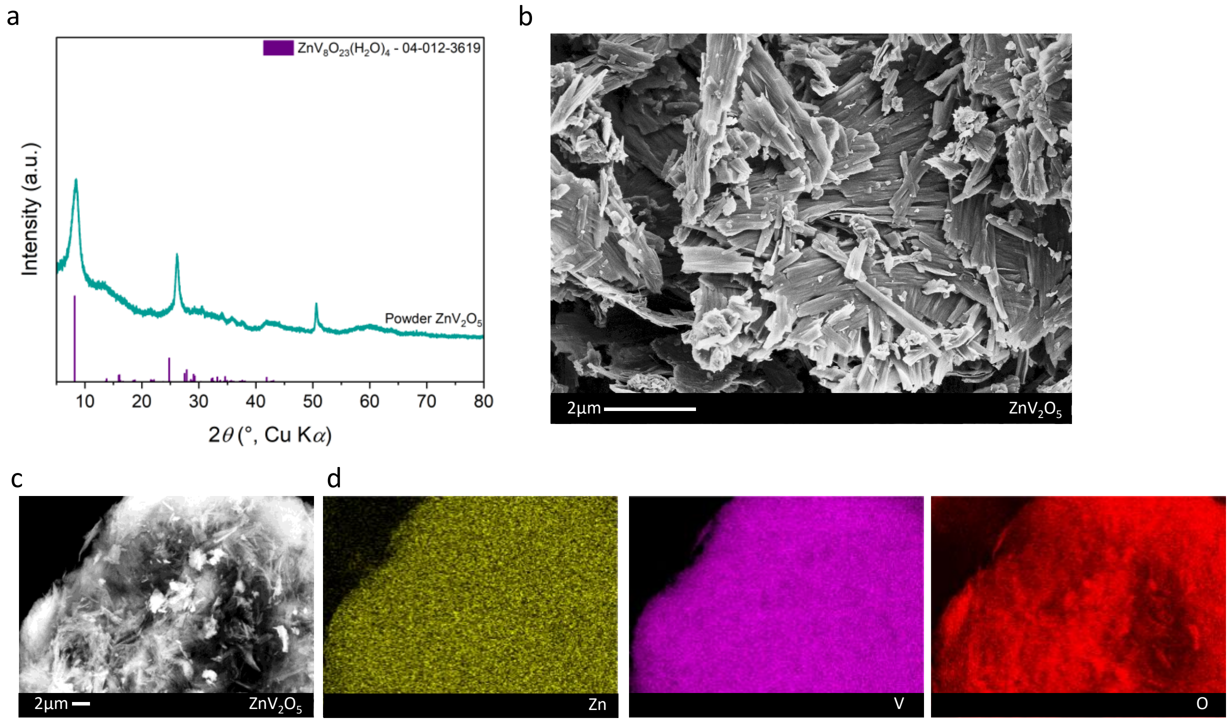

Figure S22: Compositional and morphological characterization of the as-prepared ZnV_2O_5 cathode material. a) XRD result. b) and c) SEM picture at a magnification of 10K and 3.5K respectively. d) EDX mapping of Zn, V and O.

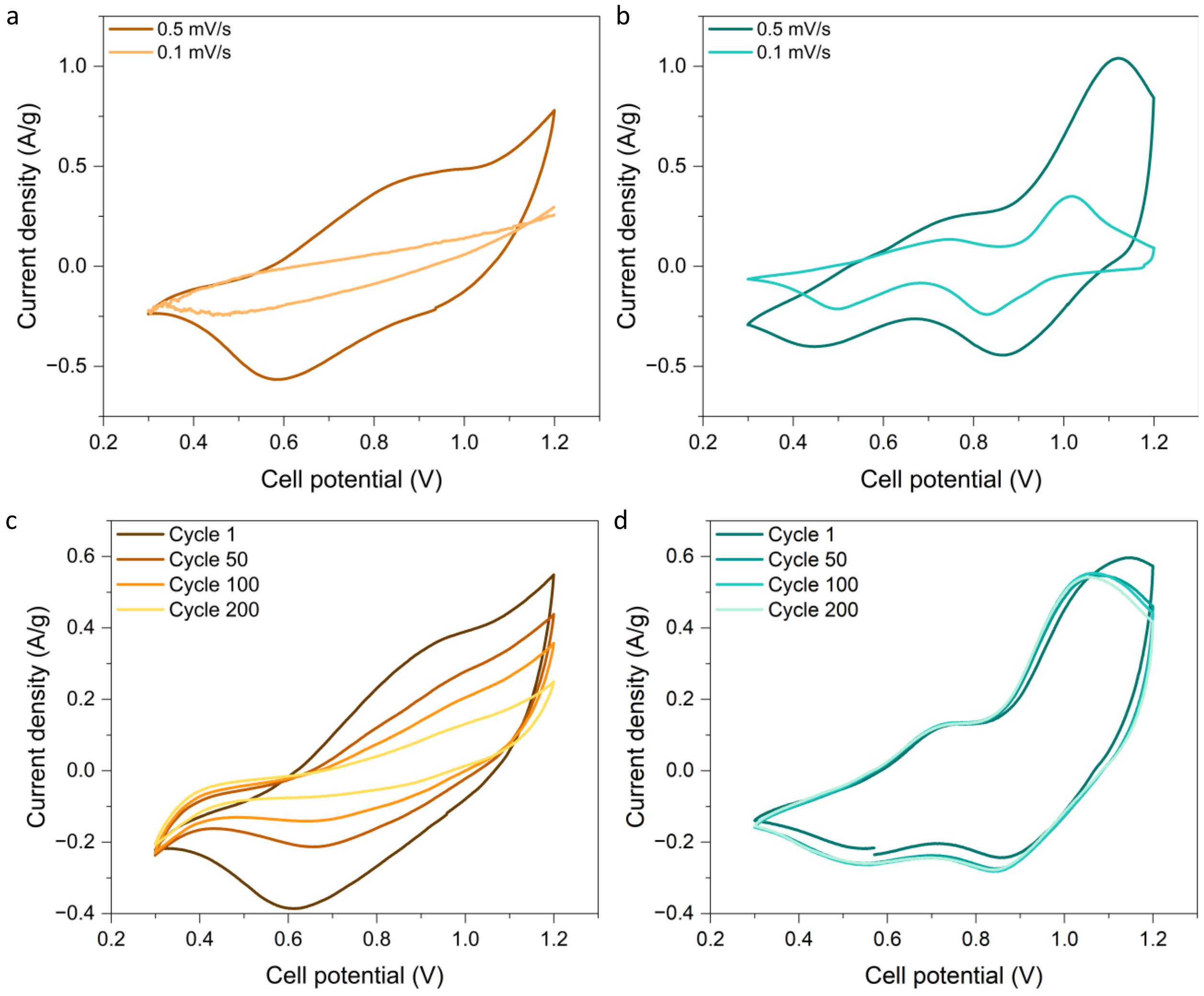

Figure S23: Cycling voltammetry of Zn||ZnVO Swagelok cells for the sat. $ZnCl_2$ and the 0.6GuaAc/1.9H_2O electrolytes. a-b) Comparison of applied current densities of 1 to 0.05 mV/s. c-d) Comparison of long-term CV cycling at 1 mV/s.

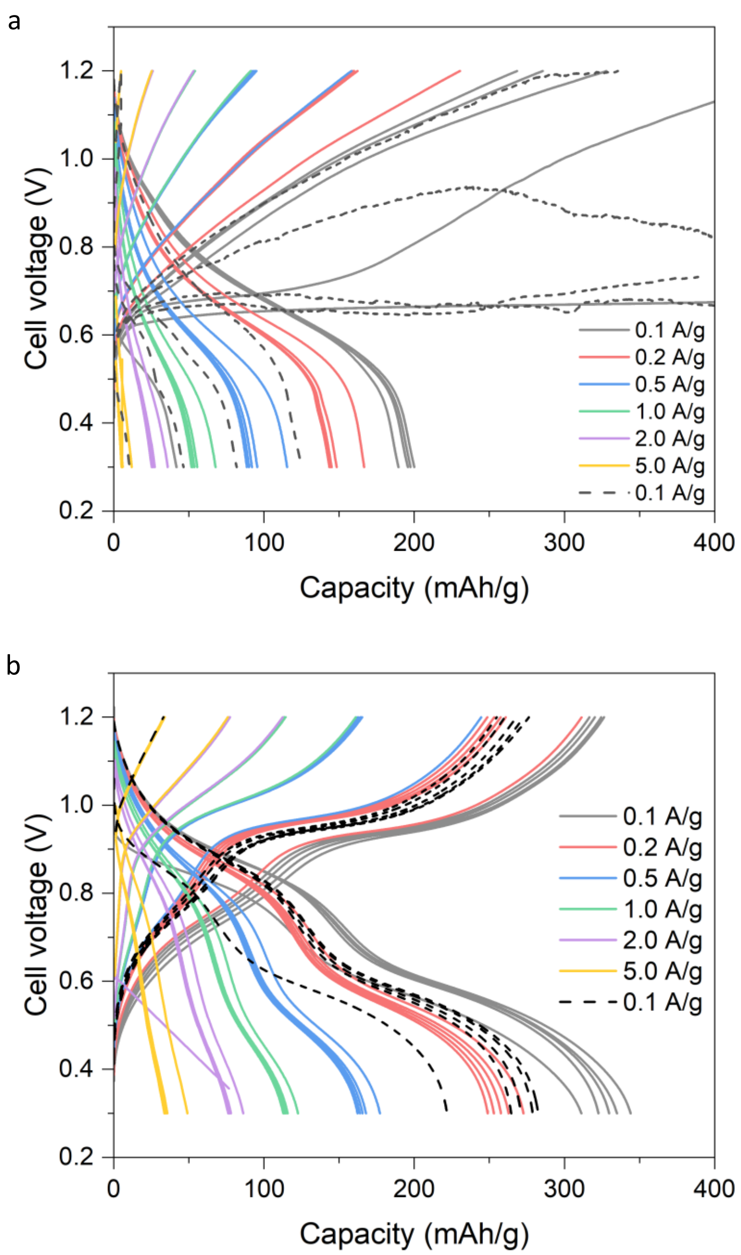

Figure S24: Rate capability profiles for the sat. $ZnCl_2$ (a) and the 0.6GuaAc/1.9H_2O electrolytes (b) for different applied current densities using ZnVO.

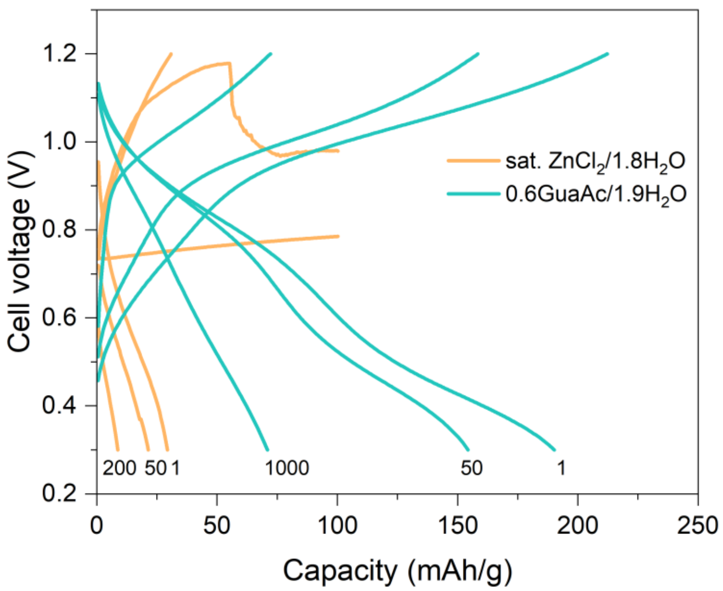

Figure S25: Profile of Zn||ZnVO long term cycling at 2 A/g using the sat. $ZnCl_2/1.8H_2O$ and the $0.6GuaAc/1.9H_2O$ electrolytes.

Supporting information tables S1-9

Table S1: Salt concentrations and pH of the $(GuaAc)_x(ZnCl_2)_{1-x}/nH_2O$ electrolytes.

Electrolyte	$C_{total\ salt}$ (m)	$C_{total\ salt}$ (g/100mL)	C_{GuaAc} (m)	C_{ZnCl2} (m)	Water/cation ratio	*pH*
sat. $ZnCl_2/1.8H_2O$	31.2	425.2	0	31.2	1.8	<0
$0.2GuaAc/2.8H_2O$	20.0	266.2	4.0	16.0	2.8	0.3
$0.4GuaAc/2.3H_2O$	24.5	316.9	9.8	14.7	2.3	2.7
$0.5GuaAc/1.4H_2O$	38.7	493.5	19.35	19.35	1.4	3.5
$0.6GuaAc/1.7H_2O$	33.5	422.4	20.1	13.4	1.7	4.9
$0.8GuaAc/2.2H_2O$	25.2	308.8	20.2	5	2.2	7.1
sat. $GuaAc/5.8H_2O$	9.6	114.6	9.6	0	5.8	9.0

Table S2: Salt concentrations and pH of the $(GuaAc)_{0.6}(ZnCl_2)_{0.4}/nH_2O$ electrolytes.

Electrolyte	$C_{total\ salt}$ (m)	$C_{total\ salt}$ (g/100mL)	C_{GuaAc} (m)	C_{ZnCl2} (m)	Water/cation ratio	*pH*
$0.6GuaAc/1.7H_2O$	33.5	422.4	20.1	13.4	1.7	4.9
$0.6GuaAc/1.8H_2O$	31.4	396.0	18.9	12.5	1.8	4.9
$0.6GuaAc/1.9H_2O$	29.6	372.7	17.8	11.8	1.9	5.0
$0.6GuaAc/2.2H_2O$	25.2	316.8	15.1	10.1	2.2	5.0

Table S3: Solid-liquid transition temperatures obtained from the DSC traces of the $(GuaAc)_X(ZnCl_2)_{X-1}/nH_2O$ electrolytes.

**Electrolyte/nH_2O**	**Tg (°C)**	**Tm (°C)**	**Tc (°C)**
sat. $ZnCl_2$/1.8H_2O	-63	27	12
0.2GuaAc/2.8H_2O	-80	-	-
0.6GuaAc/1.7H_2O	-79	-	-
0.8GuaAc/2.2H_2O	-98	-33	-48
sat. GuaAc/5.8H_2O	-105	-11	-63

Table S4: Solid-liquid transition temperatures obtained from the DSC of the $(GuaAc)_{0.6}(ZnCl_2)_{0.4}/nH_2O$ electrolytes.

**Electrolyte/nH_2O**	**Tg (°C)**	**Tm (°C)**	**Tc (°C)**
0.6GuaAc/1.7H_2O	-79	-	-
0.6GuaAc/1.8H_2O	-81	-	-
0.6GuaAc/1.9H_2O	-82	-	-
0.6GuaAc/2.2H_2O	-86	-27	-39

Table S5: Computated vibrational frequencies of different modes for a selection of species. ν = bond stretch, τ = twisting (out-of-plane bending), δs = Scissoring (in-plane-bending), ω = wagging (out-of-plane bending), subscript s = symmetric, subscript as = asymmetric.

Species	Mode	Computed vibrational frequency (cm^{-1})
**Zn_2Cl_4**	δs (Cl-Zn-Cl)+ ω($ZnCl_2$)	204<br>217
	ν_s(Zn-Cl)	262
	ν_{as}(Zn-Cl)	349
**$ZnCl_2(H_2O)_4$**	ν_s(Zn-Cl) + ν_s(Zn-OH_2)	291
**$ZnCl(H_2O)_5^+$**	ν_s(Zn-Cl) + ν_s(Zn-OH_2)	277
**$ZnCl_4^{2-}$**	ν_s(Zn-Cl)	256
**$Zn(H_2O)_6^{2+}$**	ν_s(Zn-OH_2)	334
**Ac^-**	ω(COO) + ω(CH_3)	611
	δs(O-C-O) + ω(CH_3)	649
	Deformation: δs(O-C-O) + ν(C-C)	919
	δs(CH_3) + ν_s(C-O)	1356
	δs(CH_3) + ν(C-C)+ν_s(C-O)	1408
	δs(CH_2)+ ν_{as}(C-O)	1446
	δs(CH_2) + ν(C-C)+ν_s(C-O)	1479
**$ZnAc_2$**	ω(COO) + ω(CH_3)	626<br>627
	δs(O-C-O) + ω(CH_3)	677<br>688
	Deformation: δs(O-C-O) + ν(C-C)	941<br>943
	δs(CH_3) + ν_{as}(C-O)	1332<br>1333<br>1402<br>1403
	δs(CH_2)+ ν_{as}(C-O)	1455<br>1455

	δs(CH_2)	1470<br>1470

**Gua^+**	δs(N-C-N)	496<br>514
	ν_s(C-N)	1032
	δs(NH_2) +ν_{as}(C-N)	1587<br>1591<br>1670<br>1680

**$Gua(H_2O)_3^+$**	δs(N-C-N)	530<br>563
	ν_s(C-N)	1038
	δs(NH_2) +ν_{as}(C-N)	1591<br>1614<br>1675<br>1709<br>1714

**GuaAc**	δs(N-C-N)	518<br>555
	ω(CH_3) + ω(COO)	612
	δs(O-C-O) + ω(CH_3)	658
	Deformation: δs(O-C-O) + ν(C-C)	929
	ν_s(C-N)	1035
	δs(CH_3) + ν_s(C-O)	1361
	δs(CH_3) + ν(C-C)+ν_s(C-O)	1420
	δs(CH_2)+ ν_{as}(C-O)	1445
	δs(CH_2) + ν(C-C)+ν_s(C-O)	1479
	δs(NH_2) +ν_{as}(C-N)	1598<br>1601<br>1668<br>1701<br>1702
	ν(N-H)	3204

**GuaCl**	δs(N-C-N)	501<br>519
	ν_s(C-N)	1036
	ν(N-H)	3404<br>3432

Table S6: DFT calculated binding energies of different Zn species

Species	Binding energy (kJ/mol)
$Zn(H_2O)_6^{2+}$	-117
$Zn[Cl]^{+}\cdot 5H_2O$	-127
$Zn[Cl]_2\cdot 4H_2O$	-143
$Zn[Ac]^{+}\cdot 5H_2O$	-160
$Zn[Ac]\cdot[Cl]\cdot 4H_2O$	-166
$Zn[Ac]_2\cdot 4H_2O$	-195

Table S7: Salt concentrations and pH of the electrolyte based on reaction (3).

Electrolyte	$C_{total\ salt}$ (m)	$C_{total\ salt}$ (g/100mL)	C_{GuaAc} (m)	C_{ZnCl2} (m)	Water/cation ratio	*pH*
$(Zn(Ac)_2)_{0.33}/(GuaCl)_{0.66}$	33.6	462.2	22.4	11.2	1.6	5.6

Table S8: Fitted VFT ion conductivity parameters.

Electrolyte	T_g (K)	T_0^{σ} (K)	T_g-T_0^{σ} (K)	B^{σ} (K)	E_a^{σ} (kJ/mol)	E_a^{σ} (eV)	Adj. R^2
Sat. $ZnCl_2/1.8H_2O$	210	203	7	643	5.34	0.055	0.99844
$0.6GuaAc/1.7H_2O$	194	196	-2	436	3.63	0.038	0.99967
$0.6GuaAc/1.8H_2O$	193	191	2	461	3.84	0.040	0.99991
$0.6GuaAc/1.9H_2O$	191	190	1	426	3.54	0.037	0.99998
$0.6GuaAc/2.2H_2O$	187	190	-3	426	3.54	0.037	0.99992
Sat. $GuaAc/5.8H_2O$	168	172	-4	454	3.78	0.039	0.99979

Table S9: Fitted VFT viscosity parameters.

Electrolyte	T_g (K)	T_0^{η} (K)	T_g-T_0^{η} (K)	B^{η} (K)	E_a^{σ} (kJ/mol)	E_a^{η} (eV)	Adj. R^2
Sat. $ZnCl_2/1.8H_2O$	210	201	9	758	6.30	0.065	0.99998
$0.6GuaAc/1.7H_2O$	194	209	-15	425	3.54	0.037	0.99747
$0.6GuaAc/1.8H_2O$	193	208	-15	390	3.25	0.034	0.99910
$0.6GuaAc/1.9H_2O$	191	189	3	516	4.29	0.045	0.99990
$0.6GuaAc/2.2H_2O$	187	179	8	563	4.68	0.049	0.99968
Sat. $GuaAc/5.8H_2O$	168	160	8	502	4.17	0.043	0.99991

Table S10: Fit constraints applied for peak deconvolution of C 1s, N 1s, O 1s and Cl 2p regions (X denotes a heteroatom, typically N or O). [a] Cl 2p signals are composed of doublets, $2p_{1/2}$ and $2p_{3/2}$, in a 1:2 intensity ratio and with a spin-orbit coupling of 1.60 eV. Reported values in B.E. correspond to the Cl $2p_{3/2}$ contribution.

Component	B. E. (eV)	FWHM (eV)
	**C 1s**	
C–C	284.8 ± 0.1	1.0 – 2.0
C–X	286.2 ± 0.2	1.0 – 2.0
C=X	287.8 ± 0.3	1.0 – 2.0
X–C=X	288.9 ± 0.2	1.0 – 2.0
X–C(X)–X	291.0 ± 0.2	1.0 – 2.0
	**N 1s**	
$N_{imine\text{-}like}$	398.7 ± 0.2	1.0 – 2.0
$N_{amine\text{-}like}$	400.1 ± 0.3	1.0 – 2.5
	**O 1s**	
ZnO	530.4 ± 0.2	1.0 – 2.0
C=O, O–C=O	531.9 ± 0.3	1.0 – 2.0
C–OH	533.1 ± 0.4	1.0 – 2.0
	**Cl 2p[a]**	
Cl^- (Zn–Cl)	198.9 ± 0.2	1.2 – 1.7

References

[1] C. Yang *et al.*, “All-temperature zinc batteries with high-entropy aqueous electrolyte,” *Nat. Sustain.*, vol. 6, no. March, pp. 325–335, 2023, doi: 10.1038/s41893-022-01028-x.

[2] R. Hachicha *et al.*, “Physicochemical properties and theoretical studies of novel fragile ionic liquids based on N-allyl-N,N-dimethylethylammonium cation,” 2019. doi: 10.1016/j.molliq.2019.03.166.

[3] G. Ashiotis *et al.*, “The fast azimuthal integration Python library: {\it pyFAI},” *J. Appl. Crystallogr.*, vol. 48, no. 2, pp. 510–519, Apr. 2015, doi: 10.1107/S1600576715004306.

[4] X. Yang, P. Juhas, C. L. Farrow, and S. J. L. Billinge, “xPDFsuite: an end-to-end software solution for high throughput pair distribution function transformation, visualization and analysis,” *J. Appl. Crystallogr.*, pp. 1–4, 2014, [Online]. Available: http://arxiv.org/abs/1402.3163

[5] T. R. Gengenbach, G. H. Major, M. R. Linford, and C. D. Easton, “Practical guides for x-ray photoelectron spectroscopy (XPS): Interpreting the carbon 1s spectrum,” *J. Vac. Sci. Technol. A Vacuum, Surfaces, Film.*, vol. 39, no. 1, 2021, doi: 10.1116/6.0000682.

[6] S. Tanuma, C. J. Powell, and D. R. Penn, “Calculations of electron inelastic mean free paths. IX. Data for 41 elemental solids over the 50 eV to 30 keV range,” *Surf. Interface Anal.*, vol. 43, no. 3, pp. 689–713, 2011, doi: 10.1002/sia.3522.

[7] M. J. Frisch *et al.*, “Gaussian~16 {R}evision {C}.01,” 2016.

[8] D. Bagayoko, “Density-functional thermochemistry. III. The role of exact exchange,” 1993. [Online]. Available: https://doi.org/10.1063/1.4903408

[9] R. Krishnan, J. S. Binkley, R. Seeger, and J. A. Pople, “Self-consistent molecular orbital methods. XX. A basis set for correlated wave functions,” 1980. doi: 10.1063/1.438955.

[10] D. Han *et al.*, “A non-flammable hydrous organic electrolyte for sustainable zinc batteries,” *Nat. Sustain.*, vol. 5, no. 3, pp. 205–213, 2022, doi: 10.1038/s41893-021-00800-9.

[11] L. Cao *et al.*, “Fluorinated interphase enables reversible aqueous zinc battery chemistries,” *Nat. Nanotechnol.*, vol. 16, no. 8, pp. 902–910, 2021, doi: 10.1038/s41565-021-00905-4.

[12] D. Gomez Vazquez *et al.*, “Creating water-in-salt-like environment using coordinating anions in non-concentrated aqueous electrolytes for efficient Zn batteries,” *Energy Environ. Sci.*, 2023, doi: 10.1039/d3ee00205e.
